# Haploid genetic screens identify genetic vulnerabilities to microtubule‐targeting agents

**DOI:** 10.1002/1878-0261.12307

**Published:** 2018-05-01

**Authors:** Nora M. Gerhards, Vincent A. Blomen, Merve Mutlu, Joppe Nieuwenhuis, Denise Howald, Charlotte Guyader, Jos Jonkers, Thijn R. Brummelkamp, Sven Rottenberg

**Affiliations:** ^1^ Institute of Animal Pathology Vetsuisse Faculty University of Bern Switzerland; ^2^ Division of Biochemistry The Netherlands Cancer Institute Amsterdam The Netherlands; ^3^ Division of Molecular Oncology The Netherlands Cancer Institute Amsterdam The Netherlands; ^4^ Division of Molecular Pathology The Netherlands Cancer Institute Amsterdam The Netherlands

**Keywords:** docetaxel, FBXW7, haploid screens, vinorelbine

## Abstract

The absence of biomarkers to accurately predict anticancer therapy response remains a major obstacle in clinical oncology. We applied a genome‐wide loss‐of‐function screening approach in human haploid cells to characterize genetic vulnerabilities to classical microtubule‐targeting agents. Using docetaxel and vinorelbine, two well‐established chemotherapeutic agents, we sought to identify genetic alterations sensitizing human HAP1 cells to these drugs. Despite the fact that both drugs act on microtubules, a set of distinct genes were identified whose disruption affects drug sensitivity. For docetaxel, this included a number of genes with a function in mitosis, while for vinorelbine we identified inactivation of *FBXW7*,*RB1*, and *NF2*, three frequently mutated tumor suppressor genes, as sensitizing factors. We validated these genes using independent knockout clones and confirmed *FBXW7* as an important regulator of the mitotic spindle assembly. Upon *FBXW7* depletion, vinorelbine treatment led to decreased survival of cells due to defective mitotic progression and subsequent mitotic catastrophe. We show that haploid insertional mutagenesis screens are a useful tool to study genetic vulnerabilities to classical chemotherapeutic drugs by identifying thus far unknown sensitivity factors. These results provide a rationale for investigating patient response to vinca alkaloid‐based anticancer treatment in relation to the mutational status of these three tumor suppressor genes, and could in the future lead to the establishment of novel predictive biomarkers or suggest new drug combinations based on molecular mechanisms of drug sensitivity.

**Abbreviations**
GO: Gene Ontology
MTAs: microtubule‐targeting agents
P‐gp: P‐glycoprotein
SAC: spindle assembly checkpoint

## 1 Introduction

Despite major advances in the treatment of disseminated cancers in recent years, treatment failure due to drug resistance remains a major handicap in cancer therapy. Unfortunately, there are even patients where the chosen treatment is ineffective and primarily causes side effects. Such unsuccessful treatments might result in the accumulation of pan‐resistant cancer cells and patients might lose precious time. To avoid fruitless treatments and instead provide the best regimen for an individual patient, there is an urgent need for better predictive markers which are designed to predict whether a tumor will respond to a particular treatment (Mehta *et al*., 2010). Known predictive markers include *BRCA1/2* mutations for PARP inhibitor treatment in breast and ovarian cancer (Bryant *et al*., 2005; Farmer *et al*., 2005; Tutt *et al*., 2010), *EGFR* mutations for tyrosine kinase inhibitors in non‐small cell lung cancer (Lynch *et al*., 2004; Maemondo *et al*., 2010; Paez *et al*., 2004), and estrogen receptor status for endocrine therapy of breast cancer (EBCTCG, 2005, Harris *et al*., 2007). However, these markers cover only a small fraction of all cancer treatments and patients. A better understanding of genetic vulnerabilities in human cells could pave the way for the identification of such biomarkers (Beijersbergen *et al*., 2017; Fece de la Cruz *et al*., 2015).

During the course of treatment, many patients with cancer receive chemotherapy which includes microtubule‐targeting agents (MTAs). MTAs suppress microtubule dynamics by binding to tubulin, thereby targeting proliferating cells (Stanton *et al*., 2011). Two major classes of MTAs exist: microtubule‐stabilizing drugs, such as taxanes, and microtubule‐destabilizing drugs, such as vinca alkaloids. The former compounds bind to tubulin subunits within polymerized microtubules, leading to a stabilization of the polymer and preventing its depolymerization. In contrast, vinca alkaloids bind to free tubulin, preventing the addition of free tubulin heterodimers to a growing microtubule and thereby promoting its depolymerization. The effect on microtubule stability is observed only at high drug concentrations, however (Jordan and Wilson, 2004). The clinically more relevant mode of action of these drugs is the suppression of the microtubule's dynamic turn over, inhibiting normal progression through mitosis which requires rapid assembly and disassembly of microtubules and subsequently leading to aberrant mitosis or induction of apoptosis (Chen and Horwitz, 2002; Goncalves *et al*., 2001; Hayden *et al*., 1990; Ngan *et al*., 2001).

In this study, we use docetaxel and the vinca alkaloid vinorelbine as representative drugs of both MTA classes. Docetaxel is approved by the U.S. Food and Drug Administration for the treatment of head and neck, gastric, breast, prostate, and non‐small cell lung cancer, and vinorelbine is approved for the treatment of advanced non‐small cell lung cancer [https://www.cancer.gov/about-cancer/treatment/drugs/fda-docetaxel and https://www.cancer.gov/about-cancer/treatment/drugs/vinorelbinetartrate (last accessed 31.08.2017)]. Despite the frequent use of these antimitotic drugs, primary resistance is often encountered in the clinic, and no established markers can predict MTA treatment response in patients with cancer. To date, a number of mechanisms for drug resistance have been described including increased drug efflux, deregulated apoptotic pathways, or mutations in the drug‐binding domains of tubulin subunits (O'Neill *et al*., 2011; Orr *et al*., 2003; Szakacs *et al*., 2006). The clinical relevance of these findings remains controversial, however, and they cannot explain all cases of MTA resistance and therapy failure (Borst, 2012; van Vuuren *et al*., 2015).

Recently, it has been shown that genome‐wide insertional mutagenesis screens in haploid cells can identify novel mechanisms of resistance to classical anticancer drugs like platinum salts or topoisomerase inhibitors (Planells‐Cases *et al*., 2015; Wijdeven *et al*., 2015). In this study, we applied an insertional mutagenesis‐based method to investigate gene essentiality and synthetic lethality under conditions of MTA treatment. We compared the potential vulnerabilities for both vinorelbine and docetaxel and validated the loss of three clinically relevant tumor suppressor genes (*FBXW7*,* RB1*, and *NF2* (Valverde *et al*., 2005; Welcker and Clurman, 2008; Hadfield *et al*., 2010)) as sensitizing factors for vinorelbine.

Our study demonstrates that docetaxel and vinorelbine, although both acting on microtubules, differ in the genetic vulnerabilities they exploit, and that mutations frequently observed in patients with cancer could potentially impact therapy response to a classical chemotherapeutic drug. Furthermore, our study shows that haploid insertional mutagenesis screens are useful to search for genetic vulnerabilities to classical chemotherapeutic drugs.

## 2 Materials and methods

### 2.1 Cell lines

HAP1 cells and knockout derivate cell lines were cultured in IMDM medium containing 10% fetal bovine serum, 1% penicillin‐streptomycin, and 1 mm l‐glutamine (all reagents from Gibco, Thermo Fisher Scientific Inc., Waltham, MA, USA). Monoclonal knockout cell lines were generated using CRISPR/Cas9 with the following gRNA sequences: ABCB1: 5′‐TTGGCTTGACAAGTTGTATA‐3′; FBXW7: 5′‐AAATGAAGTCTCGTTGAAAC‐3′; NF2: 5′‐CGTCACCATGGACGCCGAGA‐3′; RB1: 5′‐CAGTGTATCGGCTAGCCTAT‐3′. At early passage, independent clones were isolated from *ΔNF2* and *ΔRB1* and expanded as clone (cln) 1 and 2. Successful generation of the monoclonal knockout cells was confirmed by Sanger sequencing of the DNA.

### 2.2 Haploid genetic screens

Gene‐trap mutagenesis of wild‐type HAP1 cells was performed as described previously (Blomen *et al*., 2015). 10^8^ mutagenized HAP1 cells were seeded in 14 T175 cell culture flasks (Corning, New York, NY, USA), treated 24 h after seeding with either 4.5× IC50 of docetaxel (7 nm, Taxotere; Sanofi Aventis, Paris, France) or 6.5× IC50 of vinorelbine (16 nm, Vinorelbine; Actavis, Luxembourg, Luxembourg), as determined in nonmutagenized wild‐type HAP1 cells (see section 2.3). After 48‐h (vinorelbine) or 72‐h (docetaxel) treatment, drug‐containing medium was removed and replaced with fresh medium without drugs. On day 10, when cells displayed 70–80% confluency, cells were harvested and fixed in prewarmed BD Phosflow fix buffer I (BD Bioscience, San Jose, CA, USA) for 10 min at 37 °C. RNAse (Qiagen, Venlo, the Netherlands) treatment (100 μg·mL^−1^) was performed at 37 °C for 1 h. Subsequently, cells were stained using 10 μg·mL^−1^ propidium iodide (Life Technologies, Carlsbad, CA, USA), strained through a 40 μm cell strainer (Falcon, Corning) before at least 30 million cells with 1n DNA content were sorted on a Bio‐Rad (Hercules, CA, USA) S3 cell sorter. Genomic DNA isolation and linear amplification mediated (LAM‐)PCR were performed as described in Blomen *et al*. (2015); as well as sequencing data processing, insertion site mapping to GRCh37 human genome assembly, and subsequent analysis of sense and antisense integrations. Four independent cultured wild‐type control datasets published in Blomen *et al*. (2015), available at SRA (SRP058962, accession numbers SRX1045464, SRX1045465, SRX1045466, SRX1045467), were used for normalization. Drug‐selected screens were performed two times with individual mutagenized HAP1 batches. Analysis criteria for the identification of sensitivity candidates were *P *=* *0.01 with an effect size cutoff* *=* *1.2.

### 2.3 IC50 determination

A total of 3500 cells per well were seeded in 96‐well plates in triplicates and treated with increasing concentrations of docetaxel or vinorelbine after 24 h; 72 h later, relative numbers of viable cells in comparison with the untreated control were calculated after measuring fluorescence intensity at 560Ex/590Em nm after addition of Cell Titer Blue (Promega, Fitchburg, WI, USA) on an Enspire Multimode Plate Reader (PerkinElmer, Waltham, MA, USA), normalized to solvent control. Experiments were performed in three independent replicates, and IC50 values were calculated using graphpad prism software (GraphPad Software, San Diego, CA, USA).

### 2.4 Drug titration experiments

Drug titrations were performed in 12‐well plates, T25 and T175 flasks to find optimal screening conditions using mutagenized HAP1 cells. Cells were seeded in equal density as in the final screens, and drugs were applied after 24 h of seeding. At day 10, wells were fixed using 4% formalin and stained with 0.1% crystal violet (Merck KGaA, Darmstadt, Germany). Quantification was performed using ColonyArea Fiji plugin (Guzman *et al*., 2014; Schindelin *et al*., 2012). Quantification shown in Fig. S1 represents three independent replicates of drug titrations, performed in duplicates.

### 2.5 Validation experiments

Cells seeded in 12‐well plates at equal density as in the screens were treated after 24 h with either half, double, or the same drug concentration as used in the screens for 48 h (vinorelbine) or 72 h (docetaxel), or left untreated. Untreated wells were fixed on day 5, drug‐treated wells on day 7 or 8. Cells were treated with vincristine, vinblastine, or vindesine in an equal fashion as with vinorelbine. A total of 4000 wild‐type and 8000 *FBXW7* ^*−/−*^ DLD1 cells were seeded in six‐well plates, treated after 24 h with the same vinorelbine concentrations as have been used for HAP1 cells. After 48 h, drug‐containing medium was replaced by blank medium and colony outgrowth was determined on day 9. Experiments were repeated at least three times. Quantification was performed as described in section 2.4.

### 2.6 Gene Ontology (GO) term analysis

Gene Ontology term analysis was performed using string‐db.org with 49 potentially sensitizing docetaxel genes and 63 sensitizing vinorelbine genes (minimum required interaction score = 0.4 with databases and co‐expression as interaction sources). GO terms were ranked after false discovery rate (fdr) values and plotted for −log_10_(fdr).

### 2.7 Antibodies

Antibodies used in this study were as follows: mouse Rb (4H1, 9309, dilution 1 : 1000), rabbit Nf2 (D3S3W, 12888, dilution 1 : 1000), rabbit C‐myc (9402, dilution 1 : 800), rabbit Aurora B/AIM1 (3094, dilution 1 : 800), rabbit Mcl‐1 (4572, dilution 1 : 800) from Cell Signaling Technology, Cambridge, UK, mouse α‐Tubulin (DM1A, T9026, dilution 1 : 4000 for western blotting and 1 : 500 for immunofluorescence staining) and mouse β‐Actin (A5441, dilution 1 : 4000) from Sigma‐Aldrich (St Louis, MO, USA), mouse Cyclin B (05‐373, dilution 1 : 1000) from Millipore (Billerica, MA, USA), and mouse Aurora A (BD610939, dilution 1 : 1000) from BD Bioscience. In‐house antibodies against Cyclin E (HE‐12, dilution 1 : 5) (Sonnen *et al*., 2013), Plk1 (36‐298‐4, dilution 1 : 5) (Yamaguchi *et al*., 2005), Mps1 (3‐472‐1, dilution 1 : 5) (Stucke *et al*., 2002), and BubR1 (68‐3‐9, dilution 1 : 5) (Elowe *et al*., 2007) were described previously.

### 2.8 Western blotting

Cells were washed with PBS, lysed in RIPA buffer (50 mm Tris/HCl pH 7.4; 1% NP‐40; 0.5% Na‐deoxycholate; 0.1% SDS; 150 mm NaCl, 2 nm EDTA, 50 mm NaF) containing complete protease inhibitor cocktail (Roche, F.Hoffmann‐La Roche Ltd, Basel, Switzerland) for 30 min on ice, and cleared by centrifugation. Protein concentration was determined using Pierce BCA assay kit (Thermo Fisher Scientific Inc.) with a BSA standard curve. Before loading, protein lysates were denatured at 95 °C for 5 min in 6× SDS sample buffer. Proteins were separated by SDS/PAGE on 7.5 or 10% gels before semi‐dry transfer to 0.45 μm nitrocellulose membranes (GE Healthcare, Chalfont St Giles, UK) and blocked in 5% dry milk powder in TBS‐T (100 mm Tris, pH 7.5, 0.9% NaCl, 0.05% Tween‐20). Membranes were incubated with primary antibodies diluted in 5% BSA in TBS‐T at 4 °C over night. After washing in TBS‐T, near‐infrared labeled secondary antibodies (IRDye, Li‐Cor Biosciences, Lincoln, NE, USA, dilution 1 : 5000) were applied for 4 h at room temperature. Images were acquired using Azure c600 fluorescent imager.

Cells for protein extraction for western blots shown in Figs 4C and S6 were synchronized in M phase using 5 μm *S*‐Trityl‐L‐cysteine (Sigma‐Aldrich) and harvested by mitotic shake off before lysis in RIPA buffer.

### 2.9 qRT‐PCR

RNA extraction was performed according to the instructions of RNeasy Mini Kit (Qiagen), and cDNA was reverse transcribed with reagents of Promega using random primers. Quantitative PCR was performed using Fast Start Universal SYBR Green Master (Rox) (Roche, F.Hoffmann‐La Roche Ltd,) on 96‐well plates in AB7500 real‐time PCR cycler (Applied Biosystems, Foster City, CA, USA). Forty cycles of 95 °C for 10 s and 60 °C (*FBXW7*) or 58 °C (*HRPT*,* MYC*) for 30 s after an initial preincubation at 95 °C for 10 min were conducted. Primers were as follows: HPRT‐forward: GAAGAGCTATTGTAATGACC, HPRT‐reverse: GCGACCTTGACCATCTTTG, FBXW7‐forward: GATAGAACCCCAGTTTCAACGAGAC, FBXW7‐reverse: TGGAGGCTCTCTGAGAGGTAACCC, MYC‐forward: TACCCTCTCAACGACAGCAG, MYC‐reverse: CGTCGAGGAGAGCAGAGAAT. The relative gene expression was calculated using the 2^−ΔΔCt^ method.

### 2.10 *FBXW7* rescue

As described in section 2.9, extracted RNA from HAP1 wild‐type cells was reverse transcribed with reagents from Promega using oligo(dT) primers. Primers to amplify full‐length cDNA of *FBXW7* including restriction enzyme sites were forward: CCGGAATTCCCACCATGAATCAGGAACTGCTCTCTGTGGG and reverse: CGAGTCGACTTACTTCATGTCCACATCAAAGTCCAGC. cDNA was amplified using AccuPrime Taq High Fidelity Polymerase (Thermo Fisher Scientific Inc.), and the corresponding band was purified using QIAquick Gel Extraction kit (Qiagen) before transformation into StrataClone TOPO vector pSC‐A‐amp/kan (Agilent Technologies, Santa Clara, CA, USA) according to the manufacturer's protocol. After purification, the pSC‐A‐amp/kan‐FBXW7 and empty pBABE vectors were digested using EcoR1 and Sal1 enzymes (New England Biolabs, Ipswich, MA, USA) for 1 h at 37 °C, followed by inactivation at 65 °C for 20 min. Ligation was performed using T4 DNA Ligase (New England Biolabs) with an insert to vector ratio of 3 to 1 for 3 h at room temperature, followed by an inactivation at 65 °C for 10 min, before transformation into DH5α. Successful cloning of pBABE‐FBXW7 was confirmed by Sanger sequencing.

Fifty percent confluent phoenix retrovirus producer cells were transfected with pBABE‐FBXW7 or empty pBABE using Turbofectin transfection reagent (Origene, Rockville, MD, USA). The next day, virus‐containing supernatant was collected, filtered through a 0.45 μm filter before application to HAP1 *ΔFBXW7* target cells with Polybrene (Merck KGaA). Virus was harvested and applied to target cells on three consecutive days. 1 μg·mL^−1^ Puromycin (Gibco) was used for selection to generate stable HAP1 *ΔFBXW7 pBABE‐FBW7* and HAP1 *ΔFBXW7 pBABE* cells.

### 2.11 Growth curve

Increasing numbers of the indicated cell lines were seeded onto 96‐well plates. After 3, 4, 5, and 6 days of growth, their relative viability was measured as described in section 2.3.

### 2.12 Reversine inhibitor experiments

A total of 5000 cells were seeded per well in 12‐well plates; 24 h later, indicated drug and Reversine (Enzo Biochem, New York, NY, USA) concentrations were applied. At the indicated time points, fresh medium with or without inhibitor was applied to the wells. After 7 days, cells were fixed, stained, and quantified as described in section 2.4. Experiments were repeated three times.

### 2.13 FACS

For FACS analysis, diploid HAP1 wild‐type and *ΔFBXW7* cells were subcloned. Cells were treated for 18 h with the indicated drug concentrations and fixed in BD Phosflow fix buffer I at 37 °C for 10 min and stained with DAPI (Invitrogen, Thermo Fisher Scientific Inc.) for 10 min at room temperature. The cell cycle stages were analyzed on a BD LSR II Flow Cytometer (blue/violet laser, filter 450/50). Data were processed using flowjo software (FlowJo LLC, Ashland, OR, USA) (*n* = 2).

### 2.14 Immunofluorescence staining

Cells were grown on coverslips, treated for 18 h with the indicated drugs, and fixed with 4% formalin for 10 min at room temperature. Cells were permeabilized in 0.2% Triton X‐100, and antibodies, diluted in 10% heat‐inactivated FCS in PBS, were applied for 1 h at room temperature. DNA was stained with DAPI (Invitrogen) before mounting using DAKO mounting media. Analysis was performed on a DeltaVision Elite High Resolution Microscope system (GE Healthcare) with Olympus IX‐70 inverted microscope with a CMOS camera, 100× Olympus Objective, and softworx (Applied Precision, Issaquah, WA, USA) software. Per condition, four times 16 adjacent image fields were randomly taken for quantification. fiji software was used to process the images (Schindelin et al., 2012).

### 2.15 Live cell imaging

Diploid cells were grown on 24‐well plates and induced with CellLight Histone 2B‐GFP, BacMam 2.0 (Thermo Fisher Scientific Inc.) with PPC 50. The next day medium was refreshed, and wells were washed several times before the application of plain or drug‐containing medium. Three to 5 h later, live cell imaging was performed for up to 24 h. Images were acquired every ten minutes on the DeltaVision microscope with a 40× Olympus Objective. fiji software was used to process the images. Time‐lapse experiments have been performed four times, and combined results of all experiments are shown.

### 2.16 Correlation analysis

For the correlation of IC50 values with gene expression data from the Sanger 1001 cell line database, gene expression data from GDSC (release 6.0) were used (Yang *et al*., 2013). The log_10_[IC50] values were retrieved from supplementary table 4A from Iorio *et al*. (2016). Cell lines were clustered according to GDSC tissue descriptor 2 and validated with matching The Cancer Genome Atlas (TCGA) labels. Cell line clusters with at least six members were taken into consideration with a positive correlation and *R* ^2 ^≥ 0.2. Statistical analysis of correlation between vinorelbine log_10_[IC50] and gene expression was performed by linear regression analysis with 95% confidence interval where goodness of fit was defined by *R* ^2^ using graphpad prism software (Version 7.01).

### 2.17 Statistical analysis

Statistical analysis was performed using graphpad prism software (Version 6.05). Student's unpaired *t*‐tests were employed as appropriate. ****P <* *0.0001; ***P =* *0.0001–0.001; **P =* *0.001–0.01; *P =* *0.01–0.05, n.s. = not significant.

## 3 Results

### 3.1 Genome‐wide loss‐of‐function screens in human haploid HAP1 cells reveal genetic vulnerabilities to microtubule‐targeting agents

**Figure 1:**
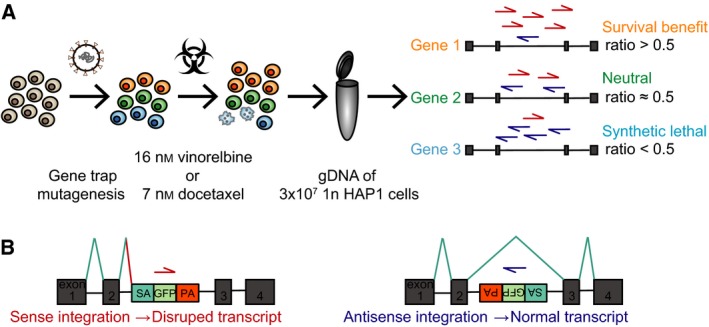
Layout of the insertional mutagenesis haploid screens. (A) Wild‐type HAP1 cells were gene‐trap mutagenized, exposed to 16 nm vinorelbine or 7 nm docetaxel, and subsequently allowed to recover until day 10. Genomic DNA of 3 × 10^7^ cells with 1n DNA content was extracted; insertion sites were amplified by LAM‐PCR before sequencing, mapping to the human genome, and normalizing to untreated cultured control datasets. Genes without effect on cellular fitness will have approximately equal numbers of disruptive sense and nondisruptive antisense integrations (ratio 0.5, neutral gene). Genes in which mutations increase survival of the cell will score with a higher proportion of sense integrations. Fewer sense integrations compared to antisense integrations will be counted in hypersensitivity genes. (B) Illustrated in a simplified fashion, intronic gene‐trap sense integration in relation to the transcriptional direction of a gene is disruptive, whereas antisense integration does not affect the function of a transcript.

To identify genes whose loss mediates sensitivity of cells to MTAs, we performed genome‐wide loss‐of‐function insertional mutagenesis screens in HAP1 cells (Fig. 1A). We aimed for a drug selection causing a mild fitness reduction of the cell population to maintain a high mutant library complexity. Based on short‐term cytotoxicity assays in which we initially determined the IC50 values of docetaxel and vinorelbine in HAP1 cells (Fig. S1A), we titrated various docetaxel and vinorelbine concentrations ranging between 2.5‐ and 6.5‐fold the IC50 (Fig. S1B). Treatment with 7 nm docetaxel for 3 days or 16 nm vinorelbine for 2 days resulted in the anticipated selection, and we subsequently used these conditions for the functional genomic screens (Fig. S1C). For this purpose, we seeded 100 million gene‐trap mutagenized cells and exposed them to docetaxel or vinorelbine. At day 10, the surviving cells were fixed and sorted for 1n DNA content, to minimize the number of diploid cells carrying heterozygous mutations. Gene‐trap insertions were identified by deep sequencing and subsequent mapping to the human genome. Four independently mutagenized wild‐type HAP1 datasets (untreated) were used as reference datasets for normalization (Blomen *et al*., 2015). All unique integration events in the genome were counted in the drug‐selected and unselected datasets. The retroviral gene‐trap cassette is unidirectional and designed to disrupt gene function upon integration in sense with the transcriptional orientation of the gene (Fig. 1B). Because mutations in genes required for fitness are detrimental to the cell, the proportion of disruptive sense integrations in a particular gene can be used as an estimate of essentiality. Comparing these proportions in a gene of interest in both unselected and drug‐selected datasets enables the identification of genes affecting cellular fitness specifically in the presence of the drugs: disruption of a gene not influencing cellular fitness under these conditions will display an approximate sense to antisense ratio of 0.5, reflecting the expected gene‐trap orientation ratio by chance. Gene‐inactivating mutations that result in a survival benefit will have a ratio > 0.5, meaning more unique disruptive sense integrations after the selection process. Finally, loss of genes causing a fitness defect will score with a ratio < 0.5, representing a loss of disruptive sense integrations. Analysis of two independent replicates for both docetaxel and vinorelbine identified several genes whose disruptions potentially confer hypersensitivity, and few genes potentially causing resistance when inactivated (Table S1).

**Figure 2:**
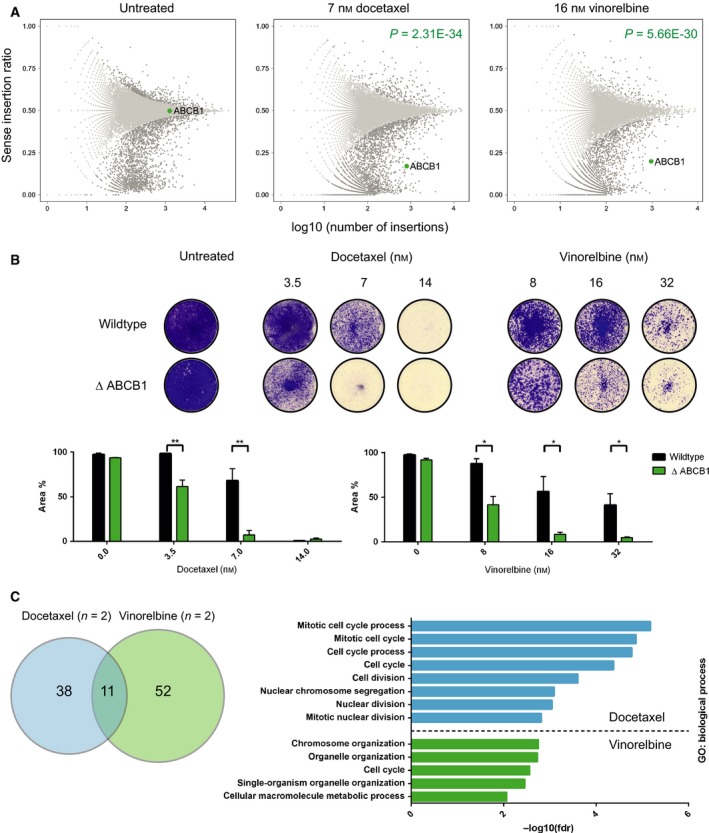
Identification of *ABCB1* validates the concept of the screens, while docetaxel and vinorelbine display different genetic vulnerabilities. (A) Unique gene‐trap insertions in *ABCB1* in untreated (*n* = 4, left panel), docetaxel‐treated (*n* = 2, panel in the center), and vinorelbine‐treated (*n* = 2, right panel) conditions. A representative example of each screening condition is shown including the *P*‐value comparing the depicted drug‐treated replicate to the depicted untreated replicate, determined by Fisher's exact *t*‐tests. *Y*‐axis represents the sense to antisense integration ratio, while log_10_ of sense/total number of insertions is plotted on the *x*‐axis. Loss of *ABCB1* was neutral in regard to cell survival without drug treatment, represented by a 0.5 sense to antisense ratio. Upon docetaxel or vinorelbine treatment, *ABCB1* was depleted for sense insertions (ratio < 0.5). (B) Validation of loss of *ABCB1* as sensitizing factor for docetaxel and vinorelbine. Equal numbers of cells were exposed to half, full, or twice the drug concentrations which have been used in the screens. Bar plots show mean quantification of three biological replicates with SEM; **P =* *0.001–0.01; *P =* *0.01–0.05. (C) Venn diagram shows overlap between vinorelbine‐ and docetaxel‐sensitizing candidates of 11 genes; 38 genes, when inactivated, sensitize uniquely to docetaxel and 52 genes to vinorelbine. Bar plot shows Gene Ontology enrichment analysis for biological processes of the sensitizing genes as −log_10_ (fdr) values. Enrichment cutoff = 0.01.

Loss of *ABCB1*, encoding for the multidrug efflux transporter P‐glycoprotein (P‐gp), was identified as one of the major sensitizing candidates in both vinorelbine and docetaxel screens (Fig. 2A). Both compounds are well‐known substrates for P‐gp (Borst, 2012; Szakacs *et al*., 2006), and we confirmed increased sensitivity toward docetaxel and vinorelbine using HAP1 cells deficient for *ABCB1* (Fig. 2B). Thus, the identification of loss of *ABCB1* as a MTA sensitizer shows that the screens identified genes that do explain drug sensitivity.

Previous haploid screening approaches identified gene disruptions that cause drug resistance by employing the density of individual disruptive gene‐trap integrations in a small pool of surviving cells as readout (Planells‐Cases *et al*., 2015; Wijdeven *et al*., 2015). As opposed to this, the layout and analysis of our current screens aimed at the identification of mutants that are absent in the surviving pool and yielded therefore potential genetic vulnerabilities. Applying stringent filtering criteria, 49 genes that, if ablated, cause sensitivity with docetaxel and 63 genes with vinorelbine were identified (Fig. 2C and Table S1). Of these, only 11 genes were shared among both treatment groups, including *ABCB1*. As both drugs affect microtubule dynamics and stability, we expected similar sensitivity profiles in both datasets. However, the genetic vulnerabilities we found were rather distinct. Although both drug screens yielded sensitizing genes associated with the gene ontology term ‘cell cycle’, only the candidates identified with docetaxel were enriched for ‘mitosis’ as expected from the exposure to a spindle poison. Some genes identified in the docetaxel screens, for example, *CCNB1*,* MAD1L1*,* MAD2L1*, or *KNTC1*, all play well‐known roles during mitosis by contributing to a functional spindle assembly checkpoint (SAC) and interact with each other (Schmit and Ahmad, 2007). In contrast to the genes that sensitize to docetaxel upon mutation, many of the vinorelbine candidates clustered under ‘chromosome/organelle organization’. Intriguingly, we found *FBXW7*,* RB1*, two genes involved in G1 cell cycle phase progression, and *NF2* among the genes which distinguished vinorelbine from docetaxel. Inactivating mutations in these genes are frequently found in various cancers and have been shown to contribute to tumorigenesis (Akhoondi *et al*., 2007; Burkhart and Sage, 2008; Cheng and Li, 2012; Petrilli and Fernandez‐Valle, 2016). We therefore validated these genes further.

### 3.2 Two clinically relevant tumor suppressor genes, *NF2* and *RB1*, show specific genetic vulnerability to vinorelbine

**Figure 3:**
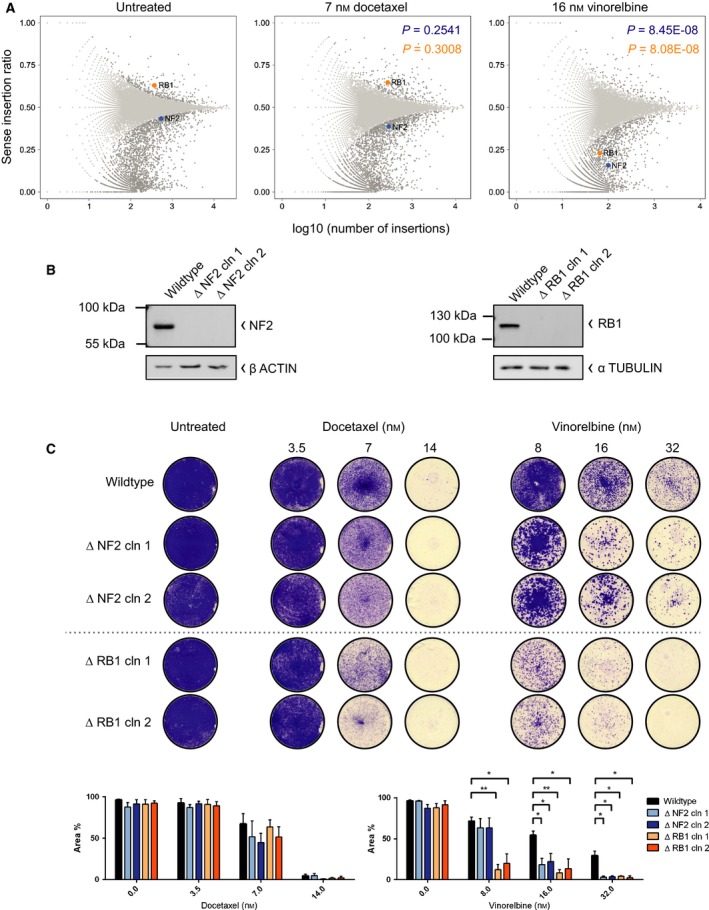
*NF2* and *RB1* score as sensitizing candidates with vinorelbine. (A) Unique gene‐trap insertions in *NF2* and *RB1* in untreated (*n* = 4, left panel), docetaxel‐treated (*n* = 2, panel in the center), and vinorelbine‐treated (*n* = 2, right panel) conditions. A representative example of each screening condition is shown including the *P*‐value comparing the depicted drug‐treated replicate to the depicted untreated replicate, determined by Fisher's exact *t*‐tests. *Y*‐axis represents the sense to antisense integration ratio, while log_10_ of sense/total number of insertions is plotted on the *x*‐axis. Loss of *RB1* scored at times with a ratio > 0.5 in untreated and docetaxel‐treated screens, indicating that disruptive sense integrations caused a survival benefit. In vinorelbine screens, a ratio < 0.5 was observed for both *RB1* and *NF2*. Noteworthy, loss of *NF2* was of slight disadvantage for survival even under untreated conditions, represented by a ratio < 0.5. (B) Confirmation of functional inactivation of *NF2* and *RB1* by western blotting with the respective antibodies (*n* = 2). (C) Validation of loss of *NF2* and *RB1* as sensitizing factor for vinorelbine with two independent knockout clones each. Equal numbers of cells were exposed to half, full, or twice the drug concentrations which have been used in the screens. Bar plots show mean quantification of three biological replicates with SEM; **P =* *0.001–0.01; *P =* *0.01–0.05.

The results of our screens indicate a sensitizing effect of *NF2* or *RB1* loss for vinorelbine in HAP1 cells (Fig. 3A). In the untreated controls, loss of *NF2* indicates a slight deficit in cellular fitness and loss of *RB1* at times gives a slight survival benefit. The same observation was made in the docetaxel screens. In contrast, both *NF2* and *RB1* sense integrations were depleted under vinorelbine treatment. To validate this finding, we tested two independent knockout clones for both *NF2* and *RB1*. In these, frameshift mutations in the first (*NF2*) and in the 20th (*RB1*) exon were introduced using CRISPR/Cas9. Successful genetic ablation was confirmed by the absence of the protein (Fig. 3B). *ΔNF2* and *ΔRB1* cells indeed displayed an increased sensitivity toward vinorelbine and vincristine treatment in comparison with wild‐type HAP1 cells (Fig. 3C and S2D). For docetaxel treatment, no significant effect on cellular survival was observed subject to the inactivation of these two genes, corroborating the results from the docetaxel screens. Thus, we verified that inactivation of *NF2* or *RB1* indeed caused hypersensitivity to vinorelbine in HAP1 cells.

### 3.3 *FBXW7* mutation is a novel genetic vulnerability to vinorelbine

**Figure 4:**
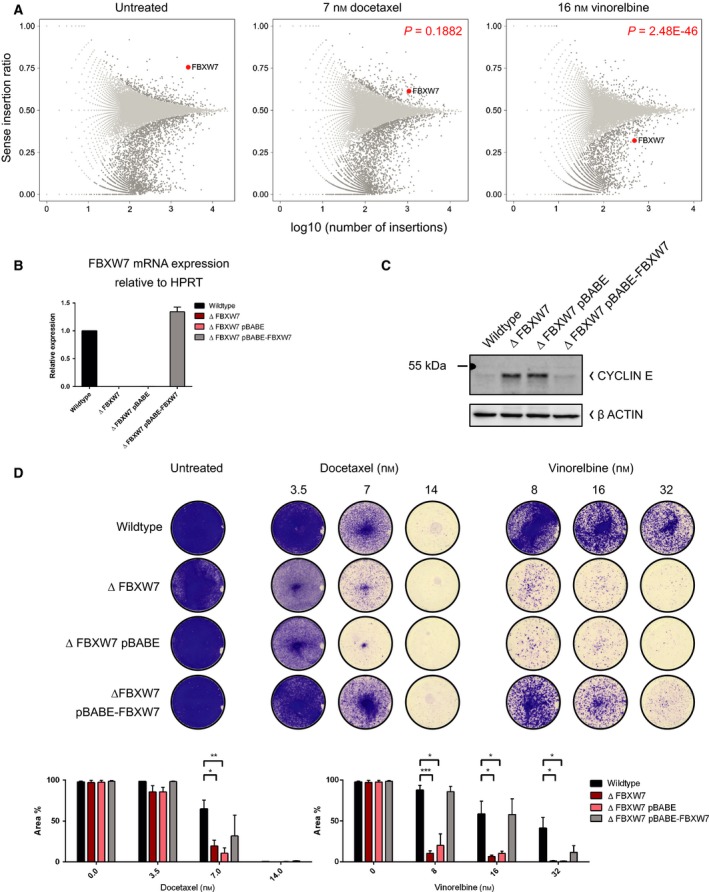
Identification of *FBXW7* mutation as a genetic vulnerability to vinorelbine. (A) Unique gene‐trap insertions in *FBXW7* in untreated (*n* = 4, left panel), docetaxel‐treated (*n* = 2, panel in the center), and vinorelbine‐treated (*n* = 2, right panel) conditions. A representative example of each screening condition is shown including the *P*‐value comparing the depicted drug‐treated replicate to the depicted untreated replicate, determined by Fisher's exact *t*‐tests. *Y*‐axis represents the sense to antisense integration ratio, while log_10_ of sense/total number of insertions is plotted on the *x*‐axis. Loss of *FBXW7* scored with a ratio > 0.5 in untreated conditions and docetaxel‐treated screens, indicating that disruptive sense integrations caused a survival benefit. For vinorelbine treatment, loss of *FBXW7* resulted in a disadvantage for survival, represented by a ratio < 0.5. (B) Confirmation of functional inactivation and restoration of *FBXW7* mRNA relative to HPRT (*n* = 3) and by (C) western blotting against CYCLIN E, a target of *FBXW7*‐mediated degradation (*n* = 3). (D) Validation of loss of *FBXW7* as sensitizing factor for vinorelbine. Restoration of *FBXW7* in *ΔFBXW7* cells reduced the sensitivity to vinorelbine close to wild‐type levels. Equal numbers of cells were exposed to half, full, or twice the drug concentrations which have been used in the screens. Bar plots show mean quantification of three biological replicates with SEM; ***P =* *0.0001–0.001; **P =* *0.001–0.01; *P =* *0.01–0.05.

In addition to inactivation of *NF2* and *RB1*, the E3 ubiquitin ligase *FBXW7* also scored as a sensitizing candidate in the vinorelbine screens (Fig. 4A), whereas under standard culture conditions loss of *FBXW7* causes a survival benefit. To confirm this finding, we generated *FBXW7*‐deficient HAP1 cells, bearing a frameshift deletion in exon 5 (shared by all three *FBXW7* isoforms α, β, γ), using CRISPR/Cas9. Successful *FBXW7* ablation was confirmed by DNA sequencing (data not shown), qRT‐PCR (Fig. 4B) and by western blotting against cyclin E (Fig. 4C), one of the ubiquitination substrates of FBXW7 (Strohmaier *et al*., 2001). To verify the specificity of our observation, we reintroduced *FBXW7* in *ΔFBXW7* cells, and the effective restoration was confirmed using the two latter assays. We found that deletion of *FBXW7* sensitizes HAP1 cells to vinorelbine, vincristine, vinblastine, and vindesine and reintroduction of *FBXW7* cDNA restores the sensitivity back to wild‐type levels (Fig. 4D and S2A–C). This finding was furthermore validated in the colorectal cancer cell line DLD1 (Fig. S2E), in which exon 5 was deleted by homologous recombination (Rajagopalan *et al*., 2004). The effect of *FBXW7* loss on docetaxel sensitivity, however, is only minor. This is also supported by the docetaxel screens, where we did not identify *FBXW7* as a significant hit (Fig. 4A). The successful validation of *FBXW7* ablation as a sensitizing determinant for vinorelbine was somewhat unexpected, as a contribution to antitubulin chemotherapeutic sensitivity has been attributed to *FBXW7* through ubiquitination of the anti‐apoptotic protein MCL‐1 (Wertz *et al*., 2011). In the HAP1 cell line, however, we did not observe an impact of *FBXW7* loss or vinorelbine treatment on MCL‐1 protein levels (Fig. S3). We therefore propose an alternative, *MCL1*‐independent impact of *FBXW7* loss on MTA treatment response.

It is noteworthy that MYC, another ubiquitination substrate of *FBXW7* (Sato *et al*., 2015), is unaffected at the mRNA and protein level by *FBXW7* genotype in HAP1 cells (Fig. S4). MYC has been ectopically expressed while generating the HAP1 cell line (Carette *et al*., 2011; Essletzbichler *et al*., 2014) and presumably does not contribute to the observed phenotype.

*ΔFBXW7* cells and wild‐type HAP1 cells proliferate at a similar rate under standard culture conditions (Fig. S5). The sensitivity of *FBXW7‐*deficient cells to vinorelbine can therefore not be attributed to an increased proliferation rate. We therefore evaluated other potential mechanisms causing sensitivity to vinorelbine.

### 3.4 Inhibition of MPS1 overcomes the drug sensitivity in *ΔFBXW7* cells

**Figure 5:**
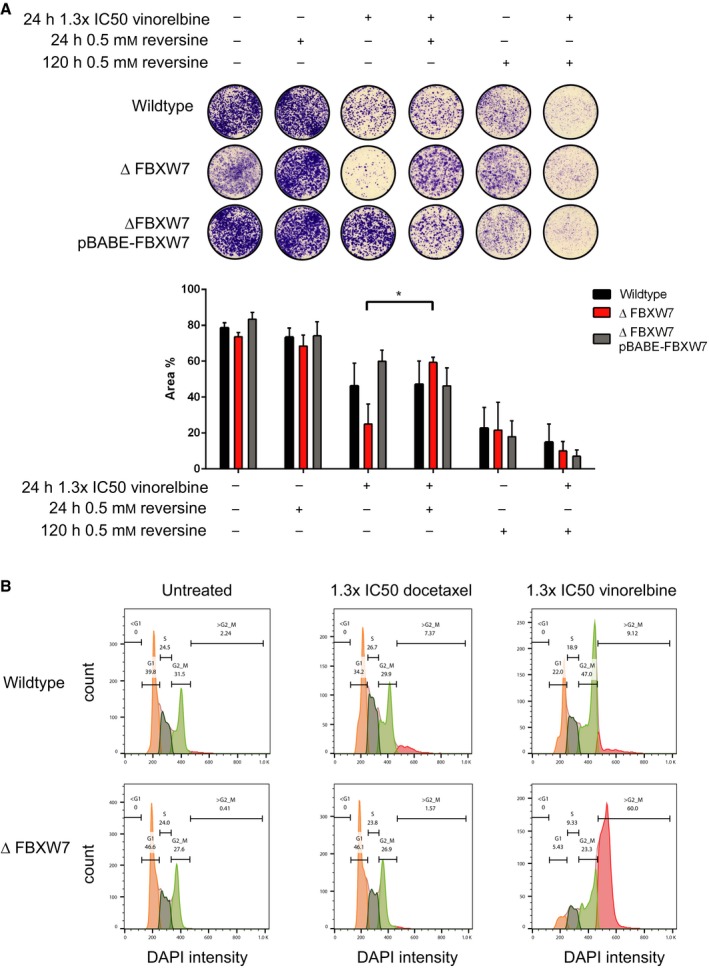
Inhibition of Mps1 by reversine protects *ΔFBXW7* cells from vinorelbine temporarily, and vinorelbine treatment causes a shift toward polyploidy in *ΔFBXW7* cells. (A) Equal numbers of cells were treated as indicated. Reversine treatment for 24 h could protect *ΔFBXW7* cells from vinorelbine hypersensitivity. Longer exposure to reversine resulted in reduced survival and increased toxicity of combined treatment with vinorelbine in all three genotypes. Bar plot shows mean quantification of three biological replicates with SEM; *P =* *0.01–0.05. (B) DNA profiles measured by DAPI intensity after 18 h of treatment with 1.3× IC50 concentrations of docetaxel or vinorelbine. Note that *ΔFBXW7* cells shift toward G2/M and polyploidy upon vinorelbine exposure (*n* = 2).

To elucidate the underlying mechanism of increased sensitivity upon FBXW7 depletion, we hypothesized that the spindle assembly checkpoint might be involved due to a recently described link between *FBXW7* and the SAC (Bailey *et al*., 2015). To test this, we inhibited the essential spindle assembly checkpoint kinase MPS1 using reversine. Inhibiting MPS1 causes mitotic progression despite improper chromosome alignment during metaphase (Hiruma *et al*., 2016; Santaguida *et al*., 2010). Reversine treatment for 24 h significantly de‐sensitized *ΔFBXW7* cells to vinorelbine (Fig. 5A) only after vinorelbine and not docetaxel treatment (Fig. S6). Prolonged exposure to reversine for 120 h decreased survival of both *FBXW7*‐proficient and *FBXW7*‐deficient cells, however. Hence, the sensitivity of *ΔFBXW7* cells to vinorelbine can temporarily be blocked by inhibiting an essential component required for proper cell division.

To further examine potential cell cycle alterations, we selected diploid HAP1 clones and analyzed their cell cycle distribution upon treatment. HAP1 cells can turn diploid during passaging or treatment through endoreduplication events (Essletzbichler *et al*., 2014) and diploid clones are more appropriate for cell cycle analysis using flow cytometry, as it is difficult to distinguish haploid G2/M from diploid G1 populations. Like the haploid cells, diploid *ΔFBXW7* cells showed increased vinorelbine sensitivity compared to diploid wild‐type cells (data not shown). When we investigated the cell cycle distribution, we found that wild‐type cells tolerated a dose of 1.3× IC50 of vinorelbine for 18 h, whereas *ΔFBXW7* cells shifted toward G2/M and polyploidy (Fig 5B). These cells also showed an increase in cell size throughout all cell cycle stages (data not shown). In contrast, exposing wild‐type or *ΔFBXW7* cells to 1.3× IC50 of docetaxel did not yield a significant impact on the cell cycle profile. To assess whether *ΔFBXW7* cells have an altered mitotic regulation, we examined some mitotic regulatory proteins by western blotting analysis of mitotic cells. We observed increased levels of CYCLIN B, MPS1, PLK1, BUBR1, AURORA A, and AURORA B in *ΔFBXW7* cells compared to wild‐type cells (Fig. S7). This is in agreement with the dependence of *FBXW7*‐deficient cells on a functional spindle assembly checkpoint and the involvement of *FBXW7* in AURORA B degradation (Bailey *et al*., 2015; Teng *et al*., 2012). Treatment with vinorelbine had no additional impact on protein levels.

### 3.5 Vinorelbine exposure reduces the number of mitotic *ΔFBXW7* cells and causes increased multinucleation and mitotic cell death

**Figure 6:**
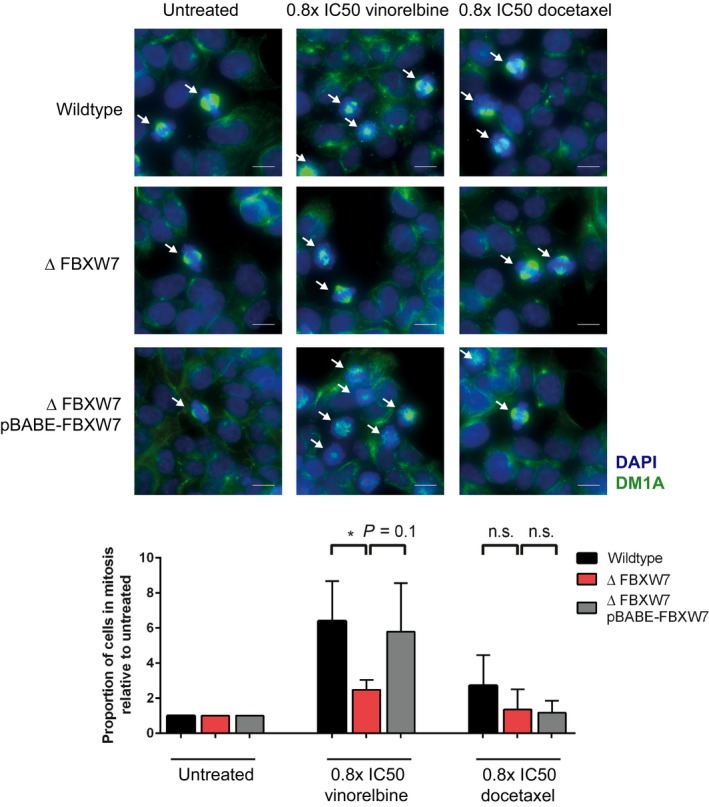
Less *ΔFBXW7* cells are in mitosis after 18 h of vinorelbine treatment. Representative images of antitubulin immunofluorescence staining after 18 h of treatment with 0.8× IC50 of vinorelbine or docetaxel. Cells in prometaphase to anaphase stage of mitosis, indicated with arrows, were counted in 4× 16 adjacent 100× power fields as percentage of total number of cells. Scale bar represents 10 μm. Mitotic cells in the three indicated cell lines, normalized to percentage of mitotic cells without treatment, of one experiment are quantified in the bar blot with SD. Note the significant lower number of mitotic cells in the *ΔFBXW7* cell line. *P =* *0.01–0.05, n.s. = not significant, *n* = 2.

To distinguish whether the >G2/M population observed in the FACS experiments contains arrested mitotic cells or polyploid/multinucleated cells, we assessed the frequency of cells in mitosis after 18‐h treatment with 0.8× IC50 of vinorelbine or docetaxel by antitubulin immunofluorescence staining. Compared to vinorelbine‐treated wild‐type cells, significantly fewer *ΔFBXW7* cells were found in mitosis (Fig. 6A). For docetaxel treatment, no significant difference in the proportion of mitotic cells in the three genotypes was observed.

**Figure 7:**
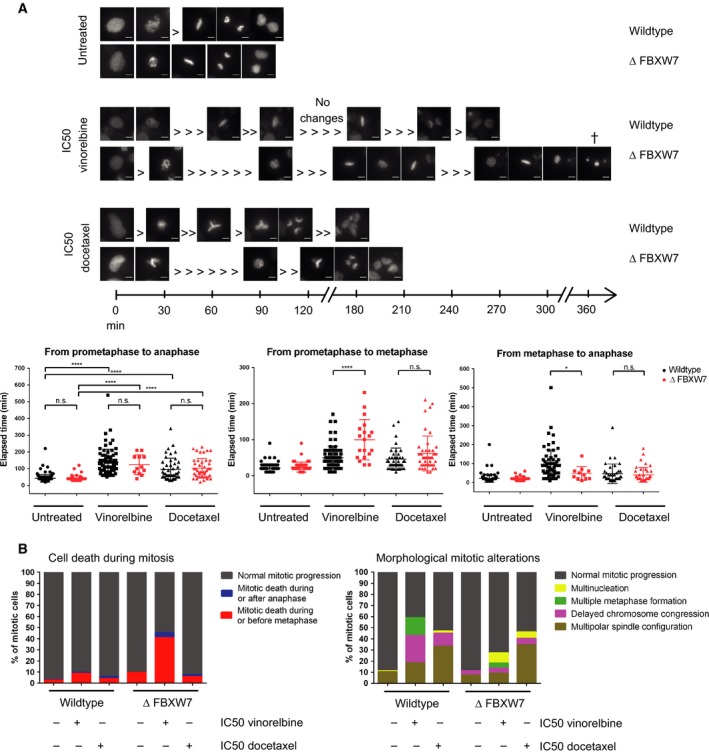
*ΔFBXW7* cells die more frequently in mitosis, undergo multinucleation, and show an increased prometaphase to metaphase time interval. (A) Representative images of H2B‐GFP‐tagged diploid wild‐type and *ΔFBXW7* cells during time‐lapse microscopy with or without MTA treatment are shown on a time line starting at the last image before entry into mitosis. Under untreated conditions, no phenotypic mitotic alterations were observed in both wild‐type and *ΔFBXW7* cells. Vinorelbine treatment caused repeatedly formation of metaphase plates in both cell lines, in the course of which *ΔFBXW7* cells died more frequently compared to wild‐type cells. Docetaxel treatment caused an increase in multipolar spindle configurations, as shown in the examples in wild‐type and *ΔFBXW7* cells. Scale bar represents 10 μm. Scatter plots show quantification of four time‐lapse experiments. Treatment with either vinorelbine or docetaxel caused a significant increase in duration of mitosis (from prometaphase to anaphase) in both cell lines. No significant difference between wild‐type and *ΔFBXW7* cells was seen in overall duration of mitosis. However, a significant prolonged prometaphase to metaphase time interval was observed in *ΔFBXW7* compared to wild‐type cells upon vinorelbine treatment, and a shorter duration of metaphase to anaphase transition. ****P <* *0.0001; *P =* *0.01–0.05, n.s. = not significant. (B) Approximately 40% of *ΔFBXW7* cells died in the course of mitosis, in particular during or before metaphase, upon vinorelbine treatment, whereas only 10% of wild‐type cells underwent cell death. Vinorelbine treatment caused repeated formations of metaphase in both wild‐type and *ΔFBXW7* cells, and frequently delayed chromosome congression in wild‐type and multinucleation in *ΔFBXW7* cells. Docetaxel treatment caused in both *ΔFBXW7* and wild‐type cell lines an increase in multipolar spindle configurations. Data are shown as combined analysis of four independent replicates.

To further decipher the mitotic alterations, we monitored GFP‐tagged H2B *ΔFBXW7* and wild‐type cells by time‐lapse microscopy. Treatment with vinorelbine or docetaxel IC50 concentrations caused a significantly prolonged mitosis in both wild‐type and *ΔFBXW7* cells (Fig. 7A). In *ΔFBXW7* cells, vinorelbine treatment resulted in a significantly extended time interval from entry into prometaphase until the metaphase plate was formed compared to wild‐type cells, while the interval from metaphase to anaphase is significantly reduced. A prolonged mitotic arrest is expected upon vinorelbine treatment (Jordan *et al*., 1998; Ngan *et al*., 2001), and we observed that wild‐type cells are able to correct the mitotic defects more frequently and undergo cell divisions resulting in viable daughter cells. In *ΔFBXW7* cells, the treatment effects are much more severe: Whereas wild‐type cells are able to repeatedly enter metaphase until all chromosomes are aligned, *ΔFBXW7* cells more frequently exit mitosis as multinucleated cells or died in the course of (repeated or single) metaphase formation (Fig. 7B). Docetaxel treatment, in contrast, mostly resulted in an increased number of multipolar spindle configurations, which was tolerated by both genotypes. Our results indicate that *ΔFBXW7* cells are unable to overcome destabilizing disturbances of the mitotic spindle caused by vinorelbine which drives the cells into mitotic catastrophe. Thus, *ΔFBXW7* cells are more effectively killed by vinca alkaloid treatment.

### 3.6 Low *FBXW7* gene expression correlates significantly with increased vinorelbine sensitivity in cell lines derived from lymphoid, thyroid, and pancreatic tumors

We next investigated whether there may be specific tumor types for which *FBXW7* could potentially serve as a useful predictive marker of vinorelbine response. For this purpose, we tested whether there is a positive correlation between *FBXW7* gene expression and vinorelbine sensitivity (log_10_[IC50] values) in the Sanger cell line dataset (Iorio *et al*., 2016). Using tissue clusters for which at least six independent cell lines were treated with vinorelbine, we found a positive correlation with *R* ^2 ^≥ 0.2 for five tumor types (‘lymphoid neoplasm other’, ‘lymphoblastic T cell leukemia’, ‘Non‐small cell lung cancer not specified’, ‘pancreas’, and ‘thyroid’) (Fig. S8A, C). Of these, linear regression analysis showed a significant correlation (*P *<* *0.05) for ‘lymphoid neoplasm other’, ‘thyroid’, and ‘pancreas’. Regarding the positive correlation between *RB1* gene expression and vinorelbine IC50 values, we identified four tissue clusters with at least six cell lines to be positively correlated with an *R* ^2 ^≥ 0.2. Of these, ‘endometrium’, ‘cervix’, and ‘lymphoid neoplasm other’ were significant (Fig. S8). For *NF2*, we did not find a tumor type with a significant correlation using the available resources (data not shown). Thus, the study of the Sanger cell line dataset supports the correlation between vinorelbine sensitivity and low *FBXW7* or *RB1* expression in tumor cell lines derived from specific tissues.

## 4 Discussion

In this study, we leveraged negative selection in mutagenized haploid cells to detect genetic vulnerabilities between genes and chemotherapeutic compounds on a genome‐wide scale. Using loss‐of‐function insertional mutagenesis screens, we identified several genes whose genetic inactivation sensitizes cells to MTAs. Although the mutational landscape found in patients is highly complex and gene‐trap‐mediated gene disruption does not reflect this entire spectrum, loss‐of‐function screening approaches are useful to gain better mechanistic insight into the problem of drug resistance. It is well known that both drugs, vinorelbine and docetaxel, are transported by P‐gp, and it is expected that *ABCB1*‐mutated cells have a survival handicap when they are exposed to these drugs. We furthermore anticipated genes around the mitotic cell cycle for both MTA screens, as inhibition of microtubule dynamics is most harmful during mitosis. For docetaxel, we indeed found a considerable number of mitotic genes, indicating that docetaxel treatment more efficiently kills cells when genes involved in mitotic regulation, in particular the spindle assembly checkpoint, are mutated. However, the genetic mutations sensitizing to vinorelbine that we identified have thus far not directly been linked to mitosis. Among these genes, we identified two genes involved in regulation of G1 cell cycle phase, *FBXW7* and *RB1* (Goodrich *et al*., 1991; Welcker and Clurman, 2008). Up to 6% of all human cancers bear mutations in *FBXW7* (Akhoondi *et al*., 2007), and *RB1* alterations occur in about 60–90% of sporadic small cell lung cancer cases, for instance (George *et al*., 2015). To our knowledge, it has not been shown thus far that loss‐of‐function mutations in *FBXW7* or *RB1* sensitize cells to vinca alkaloids. However, there are a few reports suggesting that vinca alkaloids induce a postmitotic G1 arrest, rather than a mitotic G2/M arrest (Ehrhardt *et al*., 2013; Pourroy *et al*., 2004). Moreover, we identified the disruption of another tumor suppressor gene with a microtubule‐stabilizing function, described already, *NF2* (Smole *et al*., 2014), as a specific vulnerability to vinorelbine.

Previously, it has been shown that downregulation of *FBXW7* was associated with antitubulin drug resistance due to increased levels of the anti‐apoptotic protein MCL‐1 (Wertz *et al*., 2011). In the HAP1 cell line, we did not observe a dependence of MCL‐1 protein levels on *FBXW7* genotype or vinca alkaloid treatment, however. HCT116 cells, used by Wertz *et al*. (2011), seem to be rather insensitive to MTAs compared to HAP1 cells, as treatment with 1 μm for 48 h still resulted in approximately 30% viability of cells. In another study using the same cell lines, no resistance to low concentrations of the spindle poisons nocodazole or paclitaxel was observed (Bailey *et al*., 2015). Hence, we suggest a MCL‐1‐independent effect of *FBXW7*‐mediated vinca alkaloid sensitivity at low drug concentration.

Inhibition of the spindle assembly checkpoint using reversine could temporarily protect *ΔFBXW7* cells from vinorelbine hypersensitivity. This observation confirms a previously reported dependence of *FBXW7*‐mutated cells on a functional SAC (Bailey *et al*., 2015) and supports the involvement of mitotic control mechanisms in *FBXW7*‐dependent vinorelbine sensitivity in HAP1 cells. We observed that protein levels of mitotic regulators are increased upon *FBXW7* deletion, providing more evidence for the role of FBXW7 as a regulator of mitotic processes (Teng *et al*., 2012). This is further supported by the increase of polyploidy and multinucleation in *FBXW7*‐deficient cells exposed to vinorelbine. Furthermore, a large proportion of *FBXW7*‐deficient cells died in the course of mitosis, explaining why we found less mitotic *FBXW7*‐deficient cells upon treatment. Taken together, our results suggest that upon *FBXW7* loss and vinorelbine exposure, cells more frequently fail to undergo regular mitosis, leading to mitotic death or polyploidy and multinucleation. We therefore hypothesize that *FBXW7*‐mutated tumors may benefit more from vinca alkaloid‐based MTA treatment than from taxanes and provide a rationale for MTA drug response exploration *in vivo*. Furthermore, our data indicate that vinca alkaloid treatment could be more effective when cells lack a functional G1 checkpoint, causing a stronger dependence on a functional SAC and providing a potential drug combination window for further exploration.

Our data show that two drugs targeting tubulin display different genetic vulnerabilities in HAP1 cells, indicating that their chemical–genetic interactions and cellular effects might still not have been entirely deciphered despite their longstanding clinical use. We expect that our study will provide new mechanistic insights into cellular therapy sensitivity which in the future can hopefully be leveraged to optimize microtubule‐targeting chemotherapy for patients with cancer. Unfortunately, clinical data for vinorelbine‐treated patients including gene expression and survival data are rare. However, the correlation analysis between vinorelbine sensitivity and gene expression in the Sanger cell line panel yielded specific types of tumors on which future analyses might be focused. To explore the potential of our findings as predictive biomarker for vinorelbine treatment, such a clinical study would be essential.

Previous attempts to find predictive genetic signatures for classical cytotoxic chemotherapy, for instance by correlating gene expression of cell lines to IC50 values without functional validation, yielded irreproducible results (Baggerly and Coombes, 2009). Some signatures based on patient data are, after many years, still not clinically validated (Chang *et al*., 2003; Chibon, 2013). So far, predictions have only confirmed where the therapeutic target is biologically validated, as the *BRCA1/2* PARP inhibitor example. Genome‐wide functional genomic screens provide an alternative to the *in silico* approaches and a powerful tool to identify genetic contributions to therapy response (Planells‐Cases *et al*., 2015; Steinhart *et al*., 2017; Tzelepis *et al*., 2016; Wijdeven *et al*., 2015). In an unbiased fashion, new genetic vulnerabilities of human cells to anticancer drugs can be uncovered. In our current study, we addressed drug sensitivity to classical MTAs, compounds that are still standard of care for many patients. These findings can now be translated into several cancer models and might contribute to our current repertoire of therapy response prediction and to a better understanding of the mode of action of these cytotoxic compounds.

## 5 Conclusion

In summary, our results indicate that haploid insertional mutagenesis screens are a valuable tool to study drug sensitivity. Understanding genetic vulnerabilities will be of help to optimize cancer treatment, and we present here one approach to unveil hypersensitivity to a classic chemotherapeutic drug. This could lead to the establishment of novel predictive biomarkers, result in new drug combinations, and provide deeper insight into basic biological processes of these compounds. We demonstrate that genetic vulnerabilities to classical anticancer drugs exist and that this approach gives robust results which could be confirmed using independent knockouts. We hope that our data serves as a starting point to further examine cancer vulnerabilities, in particular in *FBXW7*‐, *RB1*‐, or *NF2*‐mutated tumors.

## Author contributions

NMG planned and performed haploid screens, follow‐up experiments, and wrote the manuscript. VAB planned and analyzed haploid screens, advised on follow‐up experiments, and together with JN provided mutant clones; MM performed *in silico* analyses; DH performed follow‐up experiments; CG performed pilot haploid screens; JJ provided reagents and advised on follow‐up experiments; TRB planned, analyzed, and advised on haploid screens and follow‐up experiments; SR planned and supervised the study, analyzed data, and wrote the manuscript.

## Supporting information


**Fig. S1.** Screening conditions.
**Fig. S2.** Loss of *FBXW7* sensitizes HAP1 cells to other vinca alkaloids and *FBXW7* ^*−/−*^ DLD1 cells show increased sensitivity to vinorelbine.
**Fig. S3.** Protein levels of MCL‐1 in HAP1 cells.
**Fig. S4.** C‐MYC expression levels in *ΔFBXW7* cells.
**Fig. S5.** Growth curves of wild‐type, *ΔFBXW7*,* ΔFBXW7pBABE* and *ΔFBXW7pBABE‐FBXW7* cells
**Fig. S6.** Reversine treatment has no protective effect on docetaxel toxicity.
**Fig. S7.** *ΔFBXW7* cells have increased levels of mitotic regulatory proteins.
**Fig. S8.** Correlation of gene expression with log_10_[IC50] values for vinorelbine using the 1001 Sanger cancer cell line panel.

**Table S1.** Gene‐trap insertion counts

## References

[mol212307-bib-0001] Akhoondi S , Sun D , von der Lehr N , Apostolidou S , Klotz K , Maljukova A , Cepeda D , Fiegl H , Dafou D , Marth C *et al* (2007) FBXW7/hCDC4 is a general tumor suppressor in human cancer. Cancer Res 67, 9006–9012. 17909001 10.1158/0008-5472.CAN-07-1320

[mol212307-bib-0002] Baggerly KA and Coombes KR (2009) Deriving chemosensitivity from cell lines: forensic bioinformatics and reproducible research in high‐throughput biology. Ann Appl Stat 3, 1309–1334.

[mol212307-bib-0003] Bailey ML , Singh T , Mero P , Moffat J and Hieter P (2015) Dependence of human colorectal cells lacking the FBW7 tumor suppressor on the spindle assembly checkpoint. Genetics 201, 885–895. 26354767 10.1534/genetics.115.180653 PMC4649658

[mol212307-bib-0004] Beijersbergen RL , Wessels LFA and Bernards R (2017) Synthetic lethality in cancer therapeutics. Annu Rev Cancer Biol 1, 141–161.

[mol212307-bib-0005] Blomen VA , Majek P , Jae LT , Bigenzahn JW , Nieuwenhuis J , Staring J , Sacco R , van Diemen FR , Olk N , Stukalov A *et al* (2015) Gene essentiality and synthetic lethality in haploid human cells. Science 350, 1092–1096. 26472760 10.1126/science.aac7557

[mol212307-bib-0006] Borst P (2012) Cancer drug pan‐resistance: pumps, cancer stem cells, quiescence, epithelial to mesenchymal transition, blocked cell death pathways, persisters or what? Open Biol 2, 120066. 22724067 10.1098/rsob.120066 PMC3376736

[mol212307-bib-0007] Bryant HE , Schultz N , Thomas HD , Parker KM , Flower D , Lopez E , Kyle S , Meuth M , Curtin NJ and Helleday T (2005) Specific killing of BRCA2‐deficient tumours with inhibitors of poly(ADP‐ribose) polymerase. Nature 434, 913–917. 15829966 10.1038/nature03443

[mol212307-bib-0008] Burkhart DL and Sage J (2008) Cellular mechanisms of tumour suppression by the retinoblastoma gene. Nat Rev Cancer 8, 671–682. 18650841 10.1038/nrc2399 PMC6996492

[mol212307-bib-0009] Carette JE , Raaben M , Wong AC , Herbert AS , Obernosterer G , Mulherkar N , Kuehne AI , Kranzusch PJ , Griffin AM , Ruthel G *et al* (2011) Ebola virus entry requires the cholesterol transporter Niemann‐Pick C1. Nature 477, 340–343. 21866103 10.1038/nature10348 PMC3175325

[mol212307-bib-0010] Chang JC , Wooten EC , Tsimelzon A , Hilsenbeck SG , Gutierrez MC , Elledge R , Mohsin S , Osborne CK , Chamness GC , Allred DC *et al* (2003) Gene expression profiling for the prediction of therapeutic response to docetaxel in patients with breast cancer. Lancet 362, 362–369. 12907009 10.1016/S0140-6736(03)14023-8

[mol212307-bib-0011] Chen JG and Horwitz SB (2002) Differential mitotic responses to microtubule‐stabilizing and ‐destabilizing drugs. Cancer Res 62, 1935–1938. 11929805

[mol212307-bib-0012] Cheng Y and Li G (2012) Role of the ubiquitin ligase Fbw7 in cancer progression. Cancer Metastasis Rev 31, 75–87. 22124735 10.1007/s10555-011-9330-z

[mol212307-bib-0013] Chibon F (2013) Cancer gene expression signatures – the rise and fall? Eur J Cancer 49, 2000–2009. 23498875 10.1016/j.ejca.2013.02.021

[mol212307-bib-0014] Early Breast Cancer Trialists’ Collaborative Group (EBCTCG) (2005) Effects of chemotherapy and hormonal therapy for early breast cancer on recurrence and 15‐year survival: an overview of the randomised trials. Lancet 365, 1687–1717. 15894097 10.1016/S0140-6736(05)66544-0

[mol212307-bib-0015] Ehrhardt H , Pannert L , Pfeiffer S , Wachter F , Amtmann E and Jeremias I (2013) Enhanced anti‐tumour effects of Vinca alkaloids given separately from cytostatic therapies. Br J Pharmacol 168, 1558–1569. 23186127 10.1111/bph.12068 PMC3605866

[mol212307-bib-0016] Elowe S , Hummer S , Uldschmid A , Li X and Nigg EA (2007) Tension‐sensitive Plk1 phosphorylation on BubR1 regulates the stability of kinetochore microtubule interactions. Genes Dev 21, 2205–2219. 17785528 10.1101/gad.436007 PMC1950859

[mol212307-bib-0017] Essletzbichler P , Konopka T , Santoro F , Chen D , Gapp BV , Kralovics R , Brummelkamp TR , Nijman SM and Burckstummer T (2014) Megabase‐scale deletion using CRISPR/Cas9 to generate a fully haploid human cell line. Genome Res 24, 2059–2065. 25373145 10.1101/gr.177220.114 PMC4248322

[mol212307-bib-0018] Farmer H , McCabe N , Lord CJ , Tutt AN , Johnson DA , Richardson TB , Santarosa M , Dillon KJ , Hickson I , Knights C *et al* (2005) Targeting the DNA repair defect in BRCA mutant cells as a therapeutic strategy. Nature 434, 917–921. 15829967 10.1038/nature03445

[mol212307-bib-0019] Fece de la Cruz F , Gapp BV , Nijman SM (2015) Synthetic lethal vulnerabilities of cancer. Annu Rev Pharmacol Toxicol, 55, 513–531. 25340932 10.1146/annurev-pharmtox-010814-124511

[mol212307-bib-0020] George J , Lim JS , Jang SJ , Cun Y , Ozretic L , Kong G , Leenders F , Lu X , Fernandez‐Cuesta L , Bosco G *et al* (2015) Comprehensive genomic profiles of small cell lung cancer. Nature 524, 47–53. 26168399 10.1038/nature14664 PMC4861069

[mol212307-bib-0021] Goncalves A , Braguer D , Kamath K , Martello L , Briand C , Horwitz S , Wilson L and Jordan MA (2001) Resistance to Taxol in lung cancer cells associated with increased microtubule dynamics. Proc Natl Acad Sci U S A 98, 11737–11742. 11562465 10.1073/pnas.191388598 PMC58799

[mol212307-bib-0022] Goodrich DW , Wang NP , Qian YW , Lee EY and Lee WH (1991) The retinoblastoma gene product regulates progression through the G1 phase of the cell cycle. Cell 67, 293–302. 1655277 10.1016/0092-8674(91)90181-w

[mol212307-bib-0023] Guzman C , Bagga M , Kaur A , Westermarck J and Abankwa D (2014) ColonyArea: an ImageJ plugin to automatically quantify colony formation in clonogenic assays. PLoS One 9, e92444. 24647355 10.1371/journal.pone.0092444 PMC3960247

[mol212307-bib-0024] Hadfield KD , Smith MJ , Urquhart JE , Wallace AJ , Bowers NL , King AT , Rutherford SA , Trump D , Newman WG and Evans DG (2010) Rates of loss of heterozygosity and mitotic recombination in NF2 schwannomas, sporadic vestibular schwannomas and schwannomatosis schwannomas. Oncogene 29, 6216–6221. 20729918 10.1038/onc.2010.363

[mol212307-bib-0025] Harris L , Fritsche H , Mennel R , Norton L , Ravdin P , Taube S , Somerfield MR , Hayes DF and Bast RC Jr (2007) American Society of Clinical Oncology 2007 update of recommendations for the use of tumor markers in breast cancer. J Clin Oncol 25, 5287–5312. 17954709 10.1200/JCO.2007.14.2364

[mol212307-bib-0026] Hayden JH , Bowser SS and Rieder CL (1990) Kinetochores capture astral microtubules during chromosome attachment to the mitotic spindle: direct visualization in live newt lung cells. J Cell Biol 111, 1039–1045. 2391359 10.1083/jcb.111.3.1039 PMC2116290

[mol212307-bib-0027] Hiruma Y , Koch A , Dharadhar S , Joosten RP and Perrakis A (2016) Structural basis of reversine selectivity in inhibiting Mps1 more potently than aurora B kinase. Proteins 84, 1761–1766. 27699881 10.1002/prot.25174

[mol212307-bib-0028] Iorio F , Knijnenburg TA , Vis DJ , Bignell GR , Menden MP , Schubert M , Aben N , Goncalves E , Barthorpe S , Lightfoot H *et al* (2016) A landscape of pharmacogenomic interactions in cancer. Cell 166, 740–754. 27397505 10.1016/j.cell.2016.06.017 PMC4967469

[mol212307-bib-0029] Jordan A , Hadfield JA , Lawrence NJ and McGown AT (1998) Tubulin as a target for anticancer drugs: agents which interact with the mitotic spindle. Med Res Rev 18, 259–296. 9664292 10.1002/(sici)1098-1128(199807)18:4<259::aid-med3>3.0.co;2-u

[mol212307-bib-0030] Jordan MA and Wilson L (2004) Microtubules as a target for anticancer drugs. Nat Rev Cancer 4, 253–265. 15057285 10.1038/nrc1317

[mol212307-bib-0031] Lynch TJ , Bell DW , Sordella R , Gurubhagavatula S , Okimoto RA , Brannigan BW , Harris PL , Haserlat SM , Supko JG , Haluska FG *et al* (2004) Activating mutations in the epidermal growth factor receptor underlying responsiveness of non‐small‐cell lung cancer to gefitinib. N Engl J Med 350, 2129–2139. 15118073 10.1056/NEJMoa040938

[mol212307-bib-0032] Maemondo M , Inoue A , Kobayashi K , Sugawara S , Oizumi S , Isobe H , Gemma A , Harada M , Yoshizawa H , Kinoshita I *et al* (2010) Gefitinib or chemotherapy for non‐small‐cell lung cancer with mutated EGFR. N Engl J Med 362, 2380–2388. 20573926 10.1056/NEJMoa0909530

[mol212307-bib-0033] Mehta S , Shelling A , Muthukaruppan A , Lasham A , Blenkiron C , Laking G and Print C (2010) Predictive and prognostic molecular markers for cancer medicine. Ther Adv Med Oncol 2, 125–148. 21789130 10.1177/1758834009360519 PMC3126011

[mol212307-bib-0034] Ngan VK , Bellman K , Hill BT , Wilson L and Jordan MA (2001) Mechanism of mitotic block and inhibition of cell proliferation by the semisynthetic Vinca alkaloids vinorelbine and its newer derivative vinflunine. Mol Pharmacol 60, 225–232. 11408618 10.1124/mol.60.1.225

[mol212307-bib-0035] O'Neill AJ , Prencipe M , Dowling C , Fan Y , Mulrane L , Gallagher WM , O'Connor D , O'Connor R , Devery A , Corcoran C *et al* (2011) Characterisation and manipulation of docetaxel resistant prostate cancer cell lines. Mol Cancer 10, 126. 21982118 10.1186/1476-4598-10-126 PMC3203088

[mol212307-bib-0036] Orr GA , Verdier‐Pinard P , McDaid H and Horwitz SB (2003) Mechanisms of Taxol resistance related to microtubules. Oncogene 22, 7280–7295. 14576838 10.1038/sj.onc.1206934 PMC4039039

[mol212307-bib-0037] Paez JG , Janne PA , Lee JC , Tracy S , Greulich H , Gabriel S , Herman P , Kaye FJ , Lindeman N , Boggon TJ *et al* (2004) EGFR mutations in lung cancer: correlation with clinical response to gefitinib therapy. Science 304, 1497–1500. 15118125 10.1126/science.1099314

[mol212307-bib-0038] Petrilli AM and Fernandez‐Valle C (2016) Role of Merlin/NF2 inactivation in tumor biology. Oncogene 35, 537–548. 25893302 10.1038/onc.2015.125 PMC4615258

[mol212307-bib-0039] Planells‐Cases R , Lutter D , Guyader C , Gerhards NM , Ullrich F , Elger DA , Kucukosmanoglu A , Xu G , Voss FK , Reincke SM *et al* (2015) Subunit composition of VRAC channels determines substrate specificity and cellular resistance to Pt‐based anti‐cancer drugs. EMBO J 34, 2993–3008. 26530471 10.15252/embj.201592409 PMC4687416

[mol212307-bib-0040] Pourroy B , Carre M , Honore S , Bourgarel‐Rey V , Kruczynski A , Briand C and Braguer D (2004) Low concentrations of vinflunine induce apoptosis in human SK‐N‐SH neuroblastoma cells through a postmitotic G1 arrest and a mitochondrial pathway. Mol Pharmacol 66, 580–591. 15322250 10.1124/mol.66.3.

[mol212307-bib-0041] Rajagopalan H , Jallepalli PV , Rago C , Velculescu VE , Kinzler KW , Vogelstein B and Lengauer C (2004) Inactivation of hCDC4 can cause chromosomal instability. Nature 428, 77. 14999283 10.1038/nature02313

[mol212307-bib-0042] Santaguida S , Tighe A , D'Alise AM , Taylor SS and Musacchio A (2010) Dissecting the role of MPS1 in chromosome biorientation and the spindle checkpoint through the small molecule inhibitor reversine. J Cell Biol 190, 73–87. 20624901 10.1083/jcb.201001036 PMC2911657

[mol212307-bib-0043] Sato M , Rodriguez‐Barrueco R , Yu J , Do C , Silva JM and Gautier J (2015) MYC is a critical target of FBXW7. Oncotarget 6, 3292–3305. 25669969 10.18632/oncotarget.3203 PMC4413654

[mol212307-bib-0044] Schindelin J , Arganda‐Carreras I , Frise E , Kaynig V , Longair M , Pietzsch T , Preibisch S , Rueden C , Saalfeld S , Schmid B *et al* (2012) Fiji: an open‐source platform for biological‐image analysis. Nat Methods 9, 676–682. 22743772 10.1038/nmeth.2019 PMC3855844

[mol212307-bib-0045] Schmit TL and Ahmad N (2007) Regulation of mitosis via mitotic kinases: new opportunities for cancer management. Mol Cancer Ther 6, 1920–1931. 17620424 10.1158/1535-7163.MCT-06-0781

[mol212307-bib-3000] Smole Z , Thoma CR , Applegate KT , Duda M , Gutbrodt KL , Danuser G and Krek W (2014) Tumor suppressor NF2/Merlin is a microtubule stabilizer. Cancer Res 74, 353–362. 24282279 10.1158/0008-5472.CAN-13-1334 PMC3929585

[mol212307-bib-0046] Sonnen KF , Gabryjonczyk AM , Anselm E , Stierhof YD and Nigg EA (2013) Human Cep192 and Cep152 cooperate in Plk4 recruitment and centriole duplication. J Cell Sci 126, 3223–3233. 23641073 10.1242/jcs.129502

[mol212307-bib-0047] Stanton RA , Gernert KM , Nettles JH and Aneja R (2011) Drugs that target dynamic microtubules: a new molecular perspective. Med Res Rev 31, 443–481. 21381049 10.1002/med.20242 PMC3155728

[mol212307-bib-0048] Steinhart Z , Pavlovic Z , Chandrashekhar M , Hart T , Wang X , Zhang X , Robitaille M , Brown KR , Jaksani S , Overmeer R *et al* (2017) Genome‐wide CRISPR screens reveal a Wnt‐FZD5 signaling circuit as a druggable vulnerability of RNF43‐mutant pancreatic tumors. Nat Med 23, 60–68. 27869803 10.1038/nm.4219

[mol212307-bib-0049] Strohmaier H , Spruck CH , Kaiser P , Won KA , Sangfelt O and Reed SI (2001) Human F‐box protein hCdc4 targets cyclin E for proteolysis and is mutated in a breast cancer cell line. Nature 413, 316–322. 11565034 10.1038/35095076

[mol212307-bib-0050] Stucke VM , Sillje HH , Arnaud L and Nigg EA (2002) Human Mps1 kinase is required for the spindle assembly checkpoint but not for centrosome duplication. EMBO J 21, 1723–1732. 11927556 10.1093/emboj/21.7.1723 PMC125937

[mol212307-bib-0051] Szakacs G , Paterson JK , Ludwig JA , Booth‐Genthe C and Gottesman MM (2006) Targeting multidrug resistance in cancer. Nat Rev Drug Discov 5, 219–234. 16518375 10.1038/nrd1984

[mol212307-bib-0052] Teng CL , Hsieh YC , Phan L , Shin J , Gully C , Velazquez‐Torres G , Skerl S , Yeung SC , Hsu SL and Lee MH (2012) FBXW7 is involved in Aurora B degradation. Cell Cycle 11, 4059–4068. 23095493 10.4161/cc.22381 PMC3507501

[mol212307-bib-0053] Tutt A , Robson M , Garber JE , Domchek SM , Audeh MW , Weitzel JN , Friedlander M , Arun B , Loman N , Schmutzler RK *et al* (2010) Oral poly(ADP‐ribose) polymerase inhibitor olaparib in patients with BRCA1 or BRCA2 mutations and advanced breast cancer: a proof‐of‐concept trial. Lancet 376, 235–244. 20609467 10.1016/S0140-6736(10)60892-6

[mol212307-bib-0054] Tzelepis K , Koike‐Yusa H , de Braekeleer E , Li Y , Metzakopian E , Dovey OM , Mupo A , Grinkevich V , Li M , Mazan M *et al* (2016) A CRISPR dropout screen identifies genetic vulnerabilities and therapeutic targets in acute myeloid leukemia. Cell Rep 17, 1193–1205. 27760321 10.1016/j.celrep.2016.09.079 PMC5081405

[mol212307-bib-0055] Valverde JR , Alonso J , Palacios I and Pestana A (2005) RB1 gene mutation up‐date, a meta‐analysis based on 932 reported mutations available in a searchable database. BMC Genet 6, 53. 16269091 10.1186/1471-2156-6-53 PMC1298292

[mol212307-bib-0056] van Vuuren RJ , Visagie MH , Theron AE and Joubert AM (2015) Antimitotic drugs in the treatment of cancer. Cancer Chemother Pharmacol 76, 1101–1112. 26563258 10.1007/s00280-015-2903-8 PMC4648954

[mol212307-bib-0057] Welcker M and Clurman BE (2008) FBW7 ubiquitin ligase: a tumour suppressor at the crossroads of cell division, growth and differentiation. Nat Rev Cancer 8, 83–93. 18094723 10.1038/nrc2290

[mol212307-bib-0058] Wertz IE , Kusam S , Lam C , Okamoto T , Sandoval W , Anderson DJ , Helgason E , Ernst JA , Eby M , Liu J *et al* (2011) Sensitivity to antitubulin chemotherapeutics is regulated by MCL1 and FBW7. Nature 471, 110–114. 21368834 10.1038/nature09779

[mol212307-bib-0059] Wijdeven RH , Pang B , van der Zanden SY , Qiao X , Blomen V , Hoogstraat M , Lips EH , Janssen L , Wessels L , Brummelkamp TR *et al* (2015) Genome‐wide identification and characterization of novel factors conferring resistance to topoisomerase II poisons in cancer. Cancer Res 75, 4176–4187. 26260527 10.1158/0008-5472.CAN-15-0380

[mol212307-bib-0060] Yamaguchi T , Goto H , Yokoyama T , Sillje H , Hanisch A , Uldschmid A , Takai Y , Oguri T , Nigg EA and Inagaki M (2005) Phosphorylation by Cdk1 induces Plk1‐mediated vimentin phosphorylation during mitosis. J Cell Biol 171, 431–436. 16260496 10.1083/jcb.200504091 PMC2171270

[mol212307-bib-0061] Yang W , Soares J , Greninger P , Edelman EJ , Lightfoot H , Forbes S , Bindal N , Beare D , Smith JA , Thompson IR *et al* (2013) Genomics of Drug Sensitivity in Cancer (GDSC): a resource for therapeutic biomarker discovery in cancer cells. Nucleic Acids Res 41, D955–D961. 23180760 10.1093/nar/gks1111 PMC3531057

