# Supplementary material for: Haploid genetic screens identify genetic vulnerabilities to microtubule‐targeting agents
Source: Mol Oncol. 2018 May 1;12(6):953–71. doi: 10.1002/1878-0261.12307 (PMC5983209; doi:10.1002/1878-0261.12307)
Supplement: Supplementary file 1 — Fig. S1. Screening conditions. Fig. S2. Loss of FBXW7 sensitizes HAP1 cells to other vinca alkaloids and FBXW7 −/− DLD1 cells show increased sensitivity to vinorelbine. Fig. S3. Protein levels of MCL‐1 in HAP1 cells. Fig. S4. C‐MYC expression levels in ΔFBXW7 cells. Fig. S5. Growth curves of wild‐type, ΔFBXW7, ΔFBXW7pBABE and ΔFBXW7pBABE‐FBXW7 cells Fig. S6. Reversine treatment has no protective effect on docetaxel toxicity. Fig. S7. ΔFBXW7 cells have increased levels of mitotic regulatory proteins. Fig. S8. Correlation of gene expression with log10[IC50] values for vinorelbine using the 1001 Sanger cancer cell line panel. [file MOL2-12-953-s001.pdf]

Figure S1

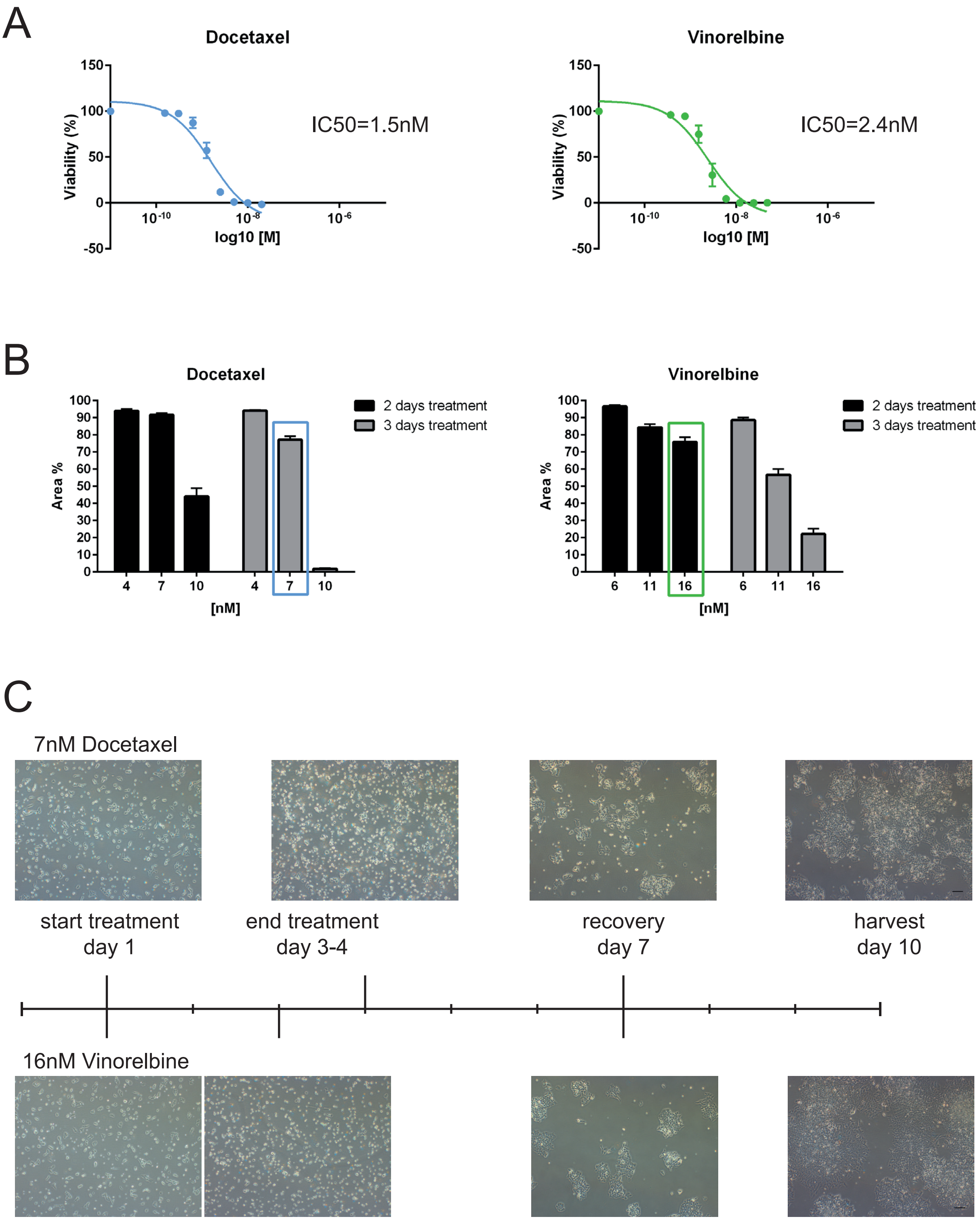

Figure S1. Screening conditions.
(A) IC50 values with cytotoxicity curves for wildtype HAP1 cells for docetaxel and vinorelbine are indicated. Mean values of n=3 experiments, performed in triplicates, are shown with SEM. (B) Quantification of drug titration experiments using gene-trap mutagenized HAP1 cells are shown as mean values of technical duplicates, n=3 with SEM. Note that different fold of IC50 values and treatment lengths for docetaxel and vinorelbine yielded an approximate 70-80% confluency on day 10. (C) Representative images are shown at the indicated time points of the screens. Scale bar represents 100μm.

Figure S2

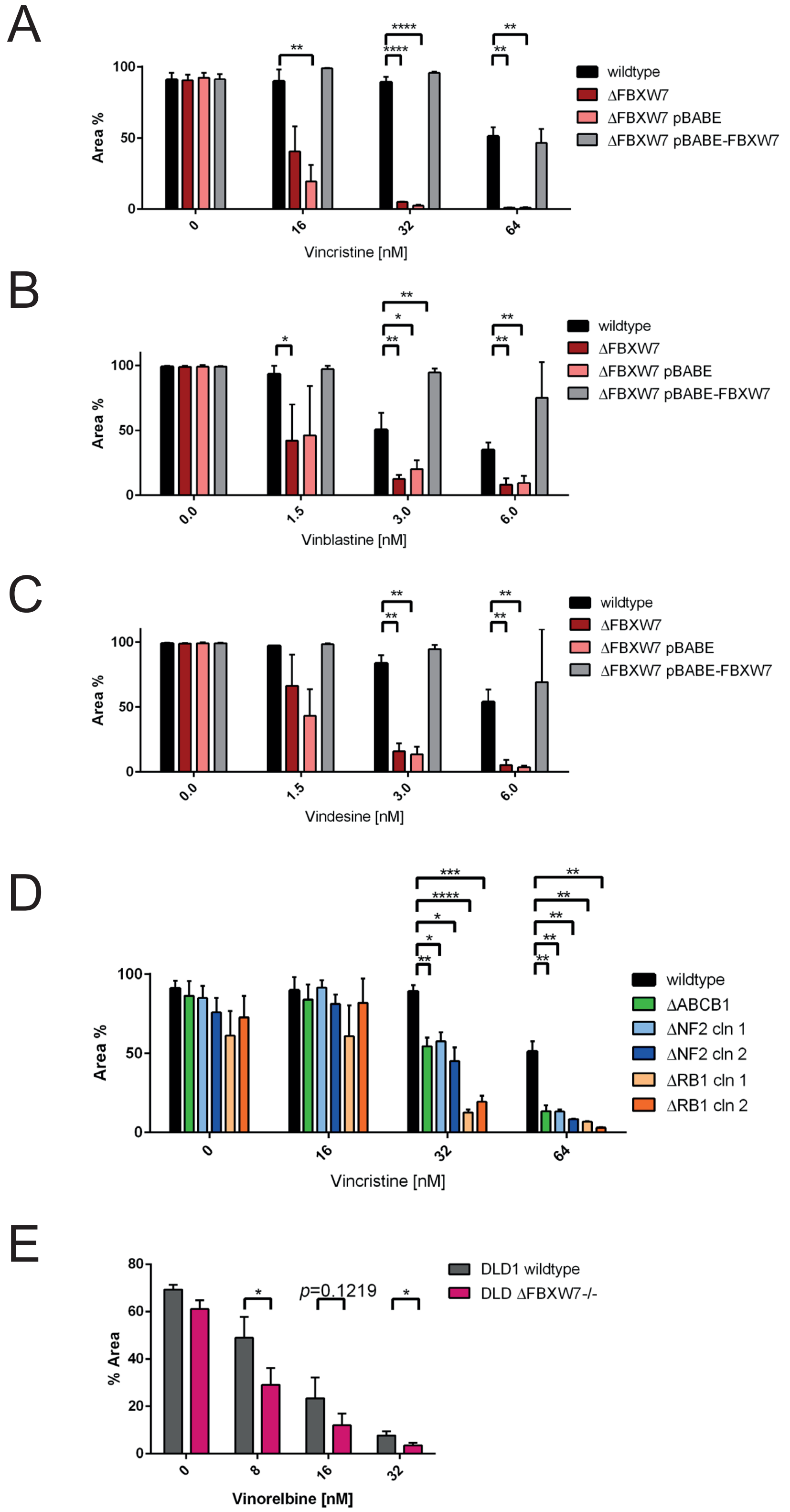

Figure S2. Loss of *FBXW7* sensitizes HAP1 cells to other vinca alkaloids and *FBXW7-/-* DLD1 cells show increased sensitivity to vinorelbine.

(A)-(C) Depletion of *FBXW7* significantly sensitized cells to vincristine, vinblastine and vindesine. (D) *ΔABCB1*, *ΔNF2* and *ΔRB1* HAP1 cells were significantly more sensitive to vincristine compared to wildtype cells. (E) *FBXW7-/-* DLD1 cells showed increased sensitivity to vinorelbine compared to wildtype DLD1 cells. Bar plots show mean quantification of three biological replicates with SEM. Graphs shown in (A) and (D) were separated in two graphs for visual reasons; **** p<0.0001; *** p=0.0001 to 0.001; ** p=0.001 to 0.01; * p=0.01 to 0.05.

Figure S3

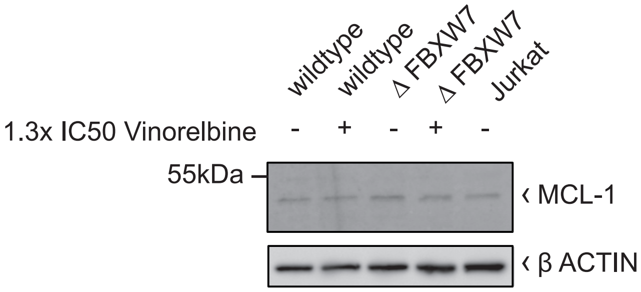

Figure S3. Protein levels of MCL-1 in HAP1 cells.
MCL-1 protein levels were determined by western blot on protein lysates of wildtype and *ΔFBXW7* cells, either treated with 1.3x IC50 vinorelbine or left untreated. Jurkat cell line lysates were blotted as control for correct molecular weight of the observed band. Note that no difference in MCL-1 protein levels was seen in relation to *FBXW7* genotype or vinorelbine treatment. n=2.

Figure S4

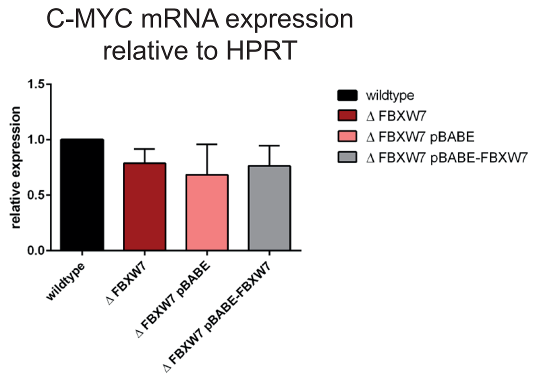

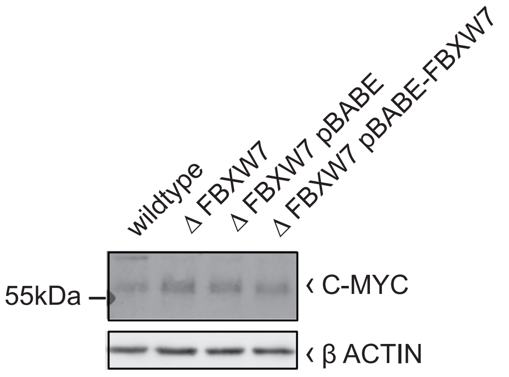

Figure S4. *C-MYC* expression levels in *ΔFBXW7* cells.
C-MYC mRNA expression levels in relation to *HPRT* were measured (n=3), and protein lysates were blotted against C-MYC (n=2). C-MYC expression levels are not affected by FBXW7 expression in HAP1 cells.

Figure S5

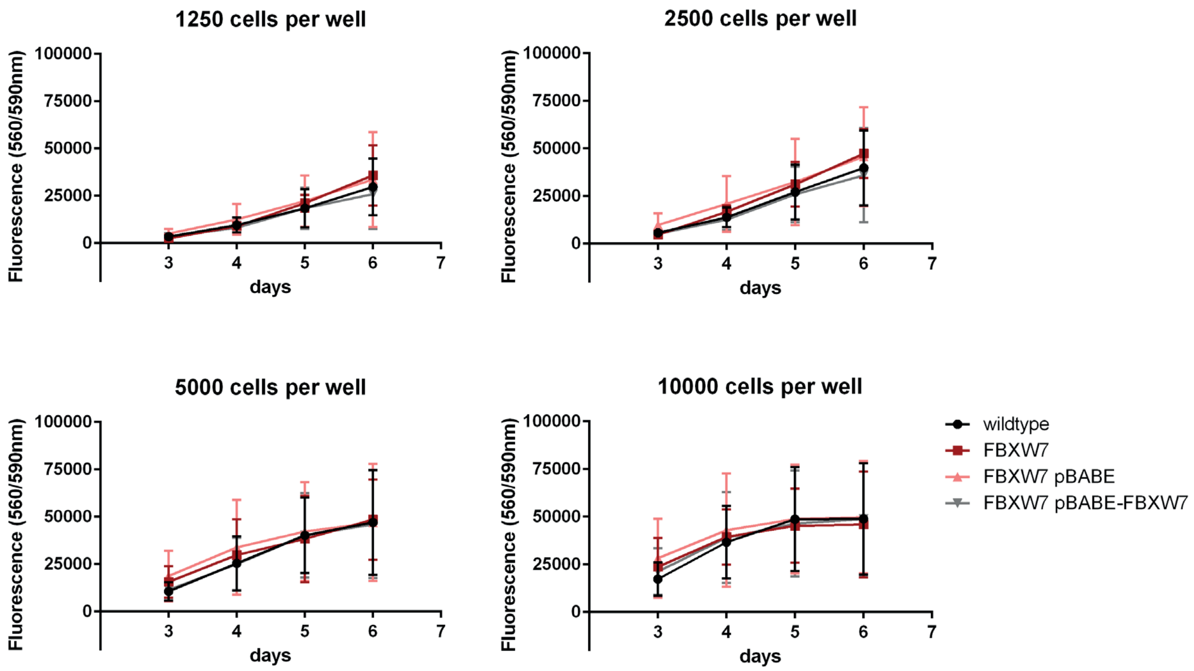

Figure S5. Growth curves of wildtype, *ΔFBXW7*, *ΔFBXW7pBABE* and *ΔFBXW7pBABE-FBXW7* cells.
Indicated cell numbers were seeded and fluorescence intensity was measured after three, four, five and six days of growth. No significant difference in growth rate in the four cell lines was observed. Results are shown as mean values of two independent replicates, performed in triplicates, with SEM.

Figure S6

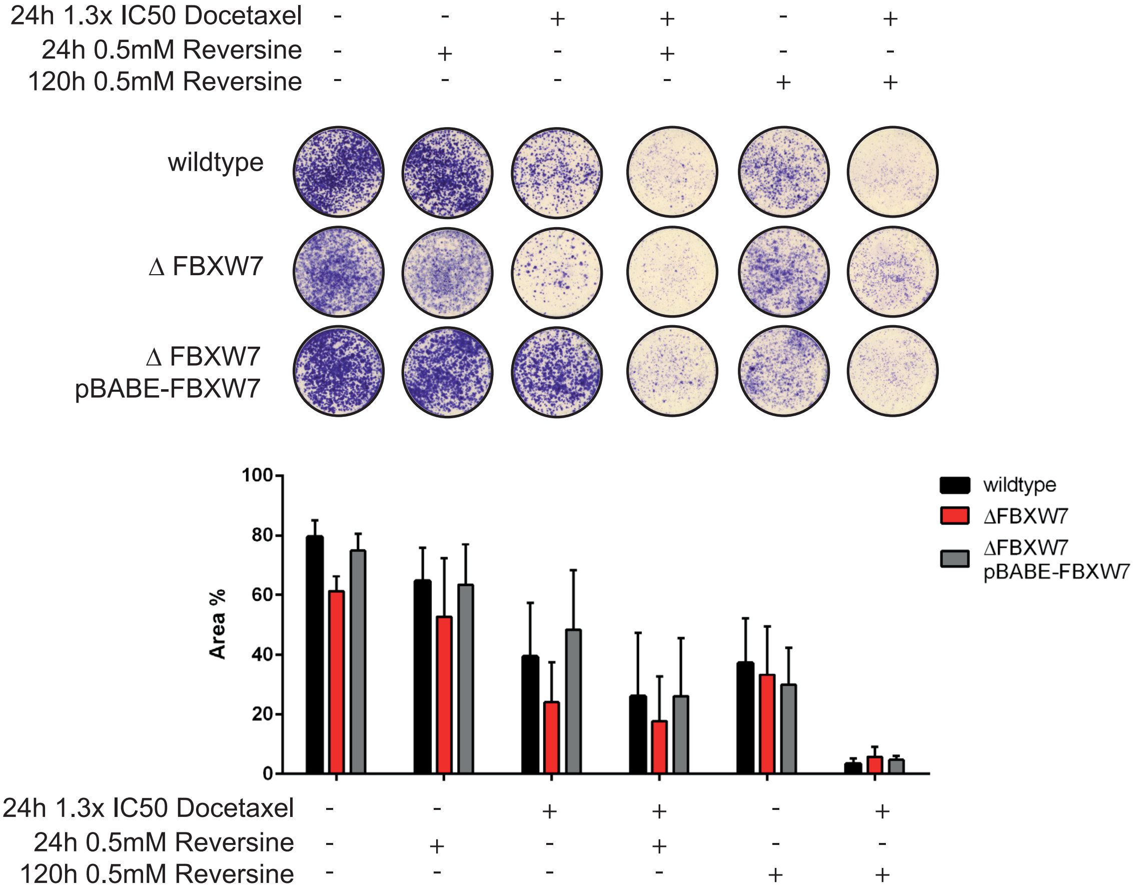

Figure S6. Reversine treatment has no protective effect on docetaxel toxicity. Equal numbers of cells were treated as indicated. Reversine treatment for 24h did not protect *ΔFBXW7* cells from docetaxel toxicity. Combination of reversine and docetaxel resulted in decreased survival in all cell lines, already after 24h treatment. Bar plot shows mean quantification of three biological replicates with SEM.

Figure S7

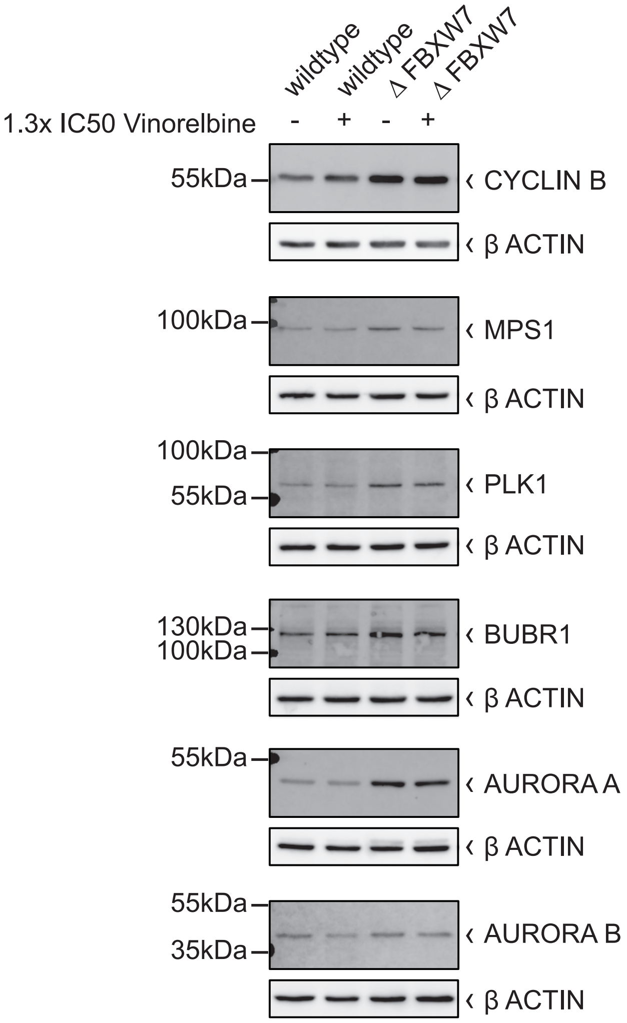

Figure S7. *ΔFBXW7* cells have increased levels of mitotic regulatory proteins. Wildtype and *ΔFBXW7* cells were treated with vinorelbine or left untreated as indicated and harvested by mitotic shake off. Vinorelbine treatment did not affect CYCLIN B, MPS1, PLK1, BUBR1, AURORA A or AURORA B protein levels in both cell lines. *ΔFBXW7* cells, however, have increased levels of all investigated mitotic proteins. n=2.

Figure S8

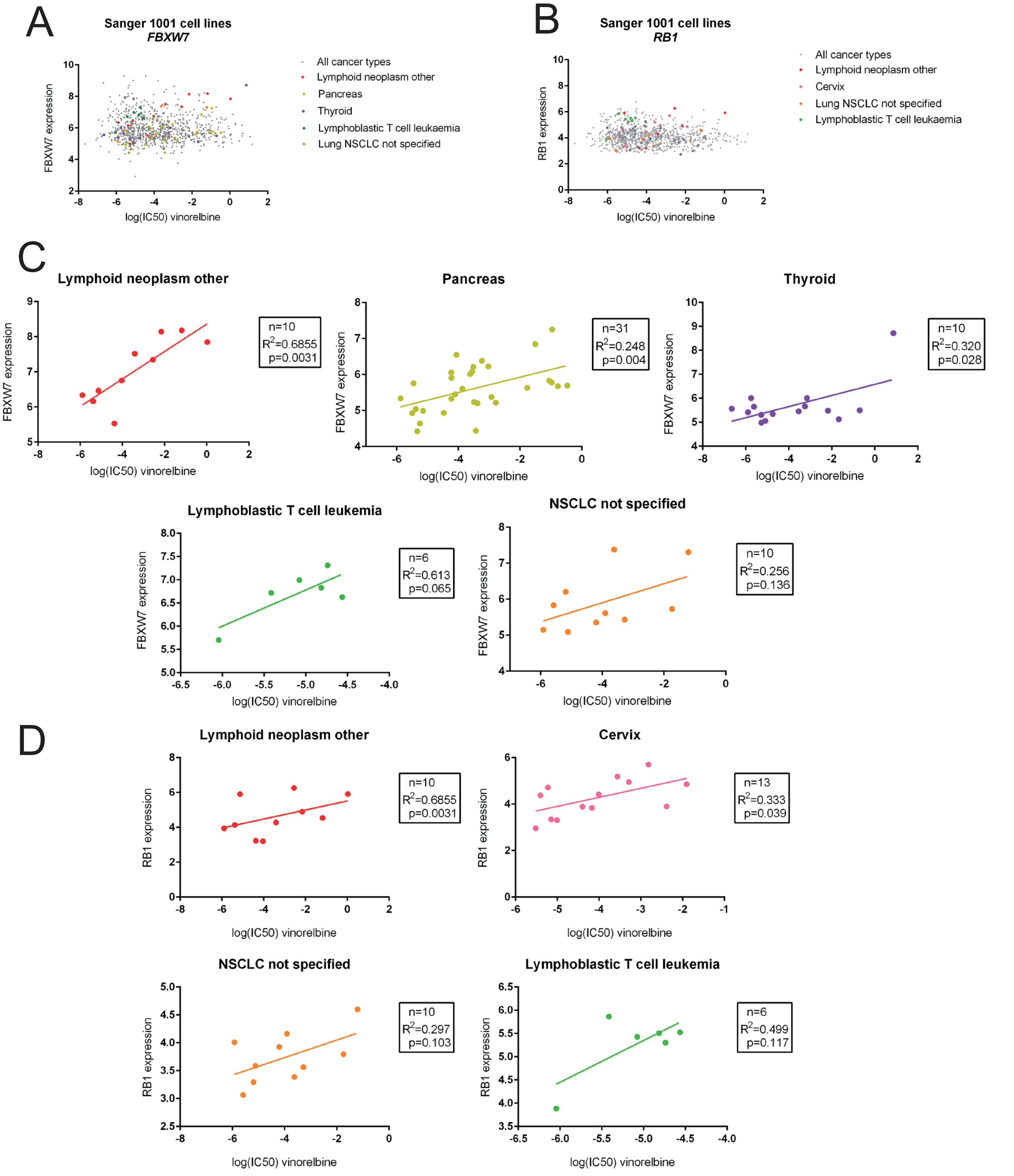

Figure S8. Correlation of gene expression with log10[IC50] values for vinorelbine using the 1001 Sanger cancer cell line panel.

Drug sensitivity data for vinorelbine was available for 867 cell lines. (A) Correlation of log10[IC50] of vinorelbine with *FBXW7* expression in all tissues based on GDSC labels. (B) Correlation of log10[IC50] of vinorelbine with *RB1* expression in all tissues based on GDSC labels. (C) Five tissue clusters containing at least six cell lines with a positive correlation and $R^2 \geq 0.2$ were analyzed. Tissue clusters "lymphoid neoplasm other", "thyroid" and "pancreas" displayed a significant correlation between low vinorelbine log10[IC50] and low *FBXW7* expression. (D) Four tissue clusters containing at least six cell lines with a positive correlation and $R^2 \geq 0.2$ were analyzed. "Cervix" and "lymphoid neoplasm other" displayed a significant correlation between low log10[IC50] and low *RB1* expression.
